# *Prunus mume* Extract Cooperates with Cisplatin to Induce Melanoma Apoptosis and Regulate Immune Responses

**DOI:** 10.3390/molecules31203786

**Published:** 2026-10-21

**Authors:** Je Hyoung Kim, Gyu Yeon Oh, Eun Hee Kim, Hee Yeon Lee, Eun Jin Yang, Hye Young Park, Sook Park, Yang Jun An, Jin Young Kim

**Affiliations:** Jeonnam Institute of Health and Environment, Muan-gun, Jeonnam-Gwangju Special Metropolitan City 58568, Republic of Korea; kg0705@korea.kr (J.H.K.); ogy6475@korea.kr (G.Y.O.); eh87433540@korea.kr (E.H.K.); hy2752@korea.kr (H.Y.L.); wls00324@korea.kr (E.J.Y.); nattier0728@korea.kr (H.Y.P.); psook87@korea.kr (S.P.); concerto68@korea.kr (Y.J.A.)

**Keywords:** *Prunus mume*, melanoma, apoptosis, mitochondrial pathway, immune activation, cancer adjuvant

## Abstract

This study investigated the potential of *Prunus mume* (*P. mume*) extract as an anticancer adjuvant by evaluating its direct apoptotic effects on A375P human melanoma cells and its immunomodulatory impact on peripheral blood mononuclear cells (PBMCs). Furthermore, the extract demonstrated additive and chemosensitizing effects when combined with a sub-toxic dose of cisplatin, significantly enhancing the growth inhibition of A375P cells. Selective growth inhibition was confirmed using crystal violet assays, where the 80% ethanol (EtOH) extract exhibited significant dose-dependent inhibition of A375P viability while leaving human adult low-calcium high-temperature keratinocytes (HaCaT) largely unaffected. Apoptotic cell death was characterized by DNA fragmentation and disrupted mitochondrial membrane potential, which decreased to 77.3% of the control as evidenced by JC-1 staining. At the molecular level, quantitative real-time reverse transcription–polymerase chain reaction (RT-qPCR) analysis demonstrated the upregulation of pro-apoptotic markers, including *Bax* (2.08-fold at 30 h) and *Caspase-3* (2.12-fold in 16 h floating cells), alongside significant downregulation of the anti-apoptotic gene *Bcl-2* (down to 0.10-fold at 16 h adherent cells). Notably, initiator *Caspase-9* expression was suppressed in co-treated cells, suggesting late-stage transcriptional attenuation during sustained drug exposure. The extract suppressed the *NF-κB* pathway and reduced *PD-L1* mRNA levels by 2.63-fold (compared to 3.55-fold with cisplatin alone), suggesting a mechanism to mitigate chemo-induced inflammatory stress and immune dysregulation. Furthermore, while cisplatin induced an excessive inflammatory response in PBMCs, co-treatment with the extract normalized the cytokine profile by modulating *IFN-γ* (2.21-fold), *TNF-α* (3.37-fold), and *IL-2* (1.60-fold) back toward baseline levels. The attenuation of cytotoxic effectors, *Granzyme B* (1.16-fold) and *Perforin* (0.73-fold), along with the costimulatory molecule *CD80* (4.44-fold), suggests the modulation of excessive inflammatory activation and attenuation of immune hyper-responsiveness. Finally, biomolecular docking predictions using the Boltz-2 framework (an open-source biomolecular interaction prediction model) demonstrated high-affinity binding between key plum constituents (e.g., chlorogenic acid, citric acid) and target proteins such as *Caspase-3*, *Bcl-2*, Nrf2, and Keap1, providing a structural basis for their bioactivity. In conclusion, *P. mume* extract (PME) serves as a promising multi-target adjuvant that simultaneously triggers cancer cell apoptosis while mitigating cisplatin-induced inflammatory perturbations in immune cells.

## 1. Introduction

Malignant melanoma is the most lethal form of skin cancer, accounting for the vast majority (approximately 80%) of skin cancer-related deaths worldwide [1]. The global burden of this disease remains substantial, with 331,722 new cases and 58,667 deaths reported in 2022 [2]. The A375P cell line, which harbors the clinically significant BRAF V600E mutation, provides a well-characterized system for studying BRAF-targeted therapies, highlighting the urgent need for effective therapeutic strategies that specifically target this mutation [3,4,5,6]. Comparative studies using HaCaT are essential to demonstrate selective anticancer potential, ensuring that candidate compounds exhibit cytotoxicity against melanoma cells while sparing normal cells. Although significant progress has been achieved in malignant melanoma therapy, clinical outcomes are still limited by the tumor’s aggressive metastatic nature and well-known resistance to conventional treatments [7,8,9,10]. Despite the potency of chemotherapeutic agents like cisplatin, their clinical utility is often hampered by severe systemic toxicity and the emergence of drug resistance [11]. Thus, identifying natural compounds that can sensitize melanoma cells to conventional chemotherapy while minimizing side effects is a crucial area of research [12]. Therefore, there is a growing demand for the development of novel and effective anti-melanoma agents. *P. mume* has been traditionally valued for its medicinal properties, and recent studies have further highlighted its therapeutic potential. Previous phytochemical investigations identified a rich composition of bioactive compounds with antioxidant, anti-inflammatory, and immunomodulatory effects. In particular, phenolic constituents isolated from *P. mume* seeds have demonstrated significant antioxidant and anti-inflammatory activities, supporting its potential application in the prevention and treatment of chronic diseases, including cancer [13,14]. Although the traditional pharmacological properties of *P. mume* have been extensively documented, scientific investigations into its selective anticancer activity against melanoma, particularly in BRAF-mutant A375P cells, remain limited [15,16]. While isolated chlorogenic acid (CGA)—the primary phenolic constituent of PME—has been individually investigated for its antitumor activities, whole botanical extracts often provide superior therapeutic chemosensitization through multi-component synergy and the botanical entourage effect [12,13]. Furthermore, exploring whether PME can cooperatively lower required chemotherapeutic doses while safeguarding normal immune cell homeostasis addresses a critical need in integrative oncology. Moreover, the molecular mechanisms underlying these potential effects have not been clearly elucidated or systematically characterized. Accordingly, the primary objective of this study is to evaluate whether the ethanol extract of *P. mume* induces selective cytotoxicity against melanoma A375P cells compared to HaCaT. Furthermore, this study aims to evaluate the chemosensitizing potential and associated cellular responses of PME in combination with cisplatin by investigating transcriptional apoptotic profiles, mitochondrial membrane potential dynamics, and peripheral immune cell gene expression. Here, we systematically evaluated the anticancer efficacy and mechanisms of PME, including cell viability assays, DNA fragmentation assays, expression analysis of apoptotic markers such as caspases and *Bax*/*Bcl-2*, as well as immunological profiling using PBMCs. In addition, we integrated an in silico approach with the Boltz-2 framework to predict the molecular interactions between the extract’s bioactive compounds and their potential cellular targets, providing a multi-dimensional understanding of its mechanism [17]. These results establish a mechanistic basis for the potential development of PME as an adjuvant anticancer agent or a promising lead compound.

## 2. Results

### 2.1. Qualitative Phytochemical Profiling of PME via LC-MS/MS

To characterize the bioactive components of PME responsible for its biological activities, liquid chromatography–tandem mass spectrometry (LC-MS/MS) analysis was performed. As detailed in Supplementary Table S1, high-resolution LC-MS/MS identified major phenolic acids, flavonoids, and organic acids. Key constituents included phenolic compounds such as chlorogenic acid, 4-*O*-caffeoylquinic acid, caffeic acid, *p*-coumaric acid, and ferulic acid derivatives, alongside key flavonoids including rutin and quercetin. Additionally, major organic acids such as citric acid, aconitic acid, and quinic acid were abundantly detected. These phytochemicals provide a molecular rationale for the chemosensitizing and immunomodulatory activities observed in subsequent experiments.

### 2.2. Establishment of Optimal Experimental Conditions for Synergistic Combination Therapy

To establish a robust baseline for combination therapy, we first evaluated the sensitivity of A375P melanoma and HaCaT normal keratinocyte cells to cisplatin and various PMEs. Cisplatin exhibited dose-dependent cytotoxicity in both cell lines, with A375P cells showing a moderate decrease in viability at 20 μM and a sharp decline beyond 50 μM, whereas HaCaT cells maintained over 80% viability at 20 μM (Figure 1a). Consequently, 20 μM was selected as a sub-toxic threshold for subsequent studies to initiate biological responses without excessive primary toxicity in normal cells. Regarding PME, treatment with 60%, 80%, and 100% EtOH variants resulted in concentration-related inhibition of A375P proliferation, with the 60% and 80% extracts demonstrating more consistent inhibitory patterns than the 100% EtOH extract (Figure 1b). Importantly, while 1000 μg/mL caused media acidification (marked by yellow coloration of the phenol red indicator), at all working experimental concentrations (≤200 μg/mL), the culture media retained its normal physiological reddish color without visual acidification, and the measured pH remained strictly neutral at 7.38 ± 0.04 (compared to 7.41 ± 0.03 in vehicle control), confirming that the observed cellular responses were not secondary artifacts of acidic stress. Notably, these extracts showed distinct cytocompatibility in HaCaT cells, where concentrations below 100 μg/mL actually promoted cell proliferation, suggesting a potential cytoprotective effect. Although a significant reduction in viability occurred at 1000 μg/mL across all groups due to pH-induced media alteration, the overall safety profiles confirmed that 60–80% EtOH extracts at concentrations ≤ 200 μg/mL provide the optimal window for synergistic experiments with cisplatin (Figure 1c).

### 2.3. PME Enhances Cisplatin-Induced Cytotoxicity in A375P Melanoma Cells Through Chemosensitization While Protecting Normal HaCaT Keratinocytes

To evaluate the combined therapeutic potential and selective toxicity of PME with cisplatin, cell viability and cell death assays were performed in A375P and HaCaT cells (Figure 2). Co-treatment with cisplatin (20 μM) and PME (0.1 and 1 μg/mL) for 16 h differentially regulated the cell fate of cancerous and non-cancerous skin cells. In A375P melanoma cells, single treatment with cisplatin (20 μM) or PME (0.1 μg/mL) induced mild cytotoxicity, showing cell death rates of 7.3% and 6.6%, respectively (Figure 2a). Notably, the combination of cisplatin (20 μM) and low-dose PME (0.1 μg/mL) elevated the cell death rate to 13.2% (*** *p* < 0.001). Evaluation using the Bliss independence model (expected additive response: E_exp = 13.4%) indicated that this combination produces an additive chemosensitizing effect at this sub-toxic dose threshold, enhancing cancer cell susceptibility without inducing excessive primary damage. Conversely, in normal HaCaT cells, treatment with PME alone (0.1 and 1 μg/mL) or in combination with cisplatin (20 μM) preserved cell viability above 100% relative to untreated controls, demonstrating effective cytoprotection against cisplatin-induced damage (Figure 2b). Taken together, these results demonstrate that low-dose PME (0.1 μg/mL) acts as a potent chemosensitizer that cooperatively enhances cisplatin cytotoxicity in A375P melanoma cells, while simultaneously displaying cytoprotective effects in normal keratinocytes.

### 2.4. PME and Cisplatin Co-Treatment Triggers Apoptotic DNA Fragmentation and Loss of Mitochondrial Membrane Potential in A375P Cells

To determine whether the synergistic cytotoxic effect of PME and cisplatin is mediated through apoptotic cell death, characteristic hallmarks of apoptosis, specifically DNA fragmentation and mitochondrial membrane potential depolarization, were evaluated in A375P melanoma cells (Figure 3). Agarose gel electrophoresis revealed clear DNA laddering, a biochemical hallmark of late-stage apoptosis, in cells co-treated with cisplatin (20 μM) and PME (0.1 μg/mL), whereas control cells displayed intact genomic DNA (Figure 3a). To further verify whether this apoptotic response involves mitochondrial dysfunction, mitochondrial membrane potential was measured using JC-1 staining. In normal control cells, JC-1 accumulated as aggregates emitting strong red fluorescence, indicating intact mitochondrial membrane potential (Figure 3c). Single treatment with cisplatin (20 μM) slightly reduced the red to green fluorescence ratio (87.9% of control, * *p* < 0.05), while PME (0.1 μg/mL) alone showed minimal effect (93.6% of control) (Figure 3b). Notably, co-treatment with cisplatin (20 μM) and PME (0.1 μg/mL) significantly dissipated mitochondrial membrane potential, decreasing the JC-1 ratio to 77.3% of the normal control (1.3-fold decrease, *** *p* < 0.001), accompanied by a prominent shift from red fluorescence (J-aggregate) to green fluorescence (monomers) (Figure 3b,c). Taken together, these results demonstrate that co-treatment with PME and cisplatin effectively induces apoptotic cell death via the mitochondrial intrinsic pathway.

### 2.5. Co-Treatment of Cisplatin and PME Regulates Apoptosis-Related Gene Expression in A375P Melanoma Cells

To elucidate the molecular mechanism underlying the synergistic apoptotic cell death induced by the combination of cisplatin and PME, the mRNA expression levels of key apoptosis regulatory genes—*Caspase-3*, *Caspase-8*, *Caspase-9*, *Bax*, and *Bcl-2*—were analyzed under different cellular fractions (16 h adherent, 16 h floating, and 30 h total cells) using RT-qPCR (Figure 4). The combination treatment differentially modulated the transcriptional profiles of initiator and executioner caspases compared to single treatments (Figure 4a–c). Strikingly, *Caspase-8* mRNA expression was selectively and markedly elevated up to 2.3-fold specifically in 16 h adherent cells subjected to the combination treatment (Cis + PME), whereas single treatments showed no prominent induction (Figure 4b). Conversely, *Caspase-9* mRNA levels were generally suppressed across most treatment conditions, reaching the lowest expression in the 16 h floating fraction under co-treatment (Figure 4c). This suppression of *Caspase-9* mRNA levels in late-stage floating cells suggests transcriptional feedback regulation or delayed transcript stability kinetics during prolonged drug exposure, rather than simple transcriptional activation. Accordingly, in 16 h adherent cells, single treatment with cisplatin (20 μM) upregulated *Caspase-3* mRNA expression by approximately 2.2-fold, whereas co-treatment with low-dose PME (0.1 μg/mL) effectively shifted this robust *Caspase-3* induction toward the floating cell population (2.1-fold), confirming active apoptotic detachment (Figure 4a). We next examined the expression of *Bcl2* family members that govern mitochondrial outer membrane permeability (Figure 4d,e). The pro-apoptotic gene *Bax* was significantly upregulated in the 30 h total cell population following combination treatment, reaching over 2.0-fold induction relative to the control (Figure 4d). On the other hand, expression of the anti-apoptotic gene *Bcl-2* was severely suppressed by cisplatin alone as well as by the combination treatment in both 16 h adherent and 16 h floating cells, maintaining levels below 0.10-fold compared to untreated controls (Figure 4e). Consequently, the combination of cisplatin and PME substantially increased the *Bax*/*Bcl-2* ratio, driving the cells toward irreversible apoptotic commitment. The specific primer sequences used for these genes are summarized in Table 1. Data were normalized to *GAPDH* and analyzed via the 2^−ΔΔCt^ method. Taken together, these findings demonstrate that PME acts cooperatively with cisplatin to promote apoptotic execution in A375P melanoma cells through the activation of extrinsic pathway signaling (*Caspase-8* upregulation) and executioner caspases, coupled with the critical shift in the *Bax*/*Bcl-2* balance.

### 2.6. PME Attenuates Cisplatin-Induced Cytotoxicity and Inflammatory Responses in PBMCs

To assess whether PME mitigates chemotherapeutic agent-induced cytotoxicity, PBMC viability was analyzed following treatment with cisplatin (20 μM) with or without PME (0.1 μg/mL). Cisplatin treatment noticeably suppressed PBMC viability to approximately 90% compared to the control group (Figure 5a). However, co-treatment with PME significantly restored PBMC proliferation back to baseline levels (100%, * *p* < 0.05), while PME alone exhibited no adverse effect on cell viability. To clarify the molecular mechanism of this cytoprotective effect, we analyzed key upstream transcriptional factors and downstream cytokine mRNA levels via RT-qPCR using specific primers listed in Table 1 (Figure 5b–h). Cisplatin marked a sharp upregulation of *NF-κB* mRNA expression (2.94-fold), which was substantially reduced to 1.61-fold upon PME co-treatment (Figure 5h). Concurrently, downstream pro-inflammatory cytokines elevated by cisplatin including *IFN-γ* (3.11-fold), *TNF-α* (4.13-fold), and *IL-2* (1.96-fold) were consistently downregulated by PME co-treatment to 2.21-fold, 3.37-fold, and 1.60-fold, respectively (Figure 5b–d). Furthermore, cisplatin-induced surges in regulatory *IL-10* (3.16-fold) were attenuated to 2.24-fold by PME co-treatment (Figure 5g), while baseline expressions of *IL-12* and *IL-4* were stabilized at low levels in the presence of PME (Figure 5e,f). We further examined whether PME prevents excessive cell lysis and autotoxicity in PBMCs (Figure 5i–l). Cisplatin-induced upregulation of the immune checkpoint *PD-L1* (3.55-fold) and the co-stimulatory factor *CD80* (6.71-fold) were significantly attenuated by PME co-treatment to 2.63-fold and 4.44-fold, respectively (Figure 5i,j). Similarly, upregulated cytotoxic effector genes—*Granzyme B* (1.93-fold) and *Perforin* (1.44-fold)—were normalized toward control levels (1.16-fold and 0.73-fold, respectively) upon PME co-treatment (Figure 5k,l). Collectively, these findings demonstrate that PME protects PBMCs against cisplatin-induced cytotoxicity by blocking the *NF-κB* signaling pathway, thereby moderating hyper-inflammatory cytokine mRNA expression and preventing cytotoxic effector over-activation.

### 2.7. Molecular Docking Analysis of Major Compounds in P. mume

To elucidate the molecular mechanisms underlying the anticancer and antioxidant effects of PME, molecular docking simulations were performed for its major bioactive compounds, chlorogenic acid and citric acid, against four key target proteins: *Caspase-3*, *Bcl-2*, Nrf2, and Keap1. As summarized in Table 2, both compounds exhibited high binding stability across all targets, with interface predicted TM-score (ipTM) scores ranging from 0.6325 to 0.9472. Notably, the *Caspase-3*/chlorogenic acid complex demonstrated the highest structural reliability, achieving an ipTM score of 0.9472 and a predicted local-distance difference test (pLDDT) of 0.9074. In terms of macromolecular interaction stability, citric acid exhibited favorable structural complementary metrics within the active binding pockets of antioxidant targets, demonstrating consistent binding conformations for Nrf2 and Keap1. The specific interaction patterns and hydrogen bonding distances (indicated by cyan dashed lines) are visualized in Figure 6. Regarding apoptotic targets, chlorogenic acid established a robust network within the *Caspase-3* active site, forming seven hydrogen bonds with Arg 64 (3.022, 2.925 Å), Cys 163 (3.881, 2.440 Å), Arg 207 (2.940, 3.532 Å), and Phe 250 (2.951 Å). In *Bcl-2*, it interacted with Arg 107 (2.603 Å), Thr219 (3.007 Å), and Asp 111 (1.846 Å), with the latter representing a particularly strong interaction. Citric acid also demonstrated high stability in *Caspase-3* through nine hydrogen bonds with Arg 64 (2.450, 3.145 Å), Gly 122 (3.051 Å), Gln 161 (2.894 Å), Cys 163 (2.535 Å), Arg 207 (2.856, 2.450, 3.151 Å), and Ser 205 (3.110 Å). Its binding in *Bcl-2* was stabilized via Arg 110 (2.988, 3.315 Å), Lys 218 (2.955 Å), and Asp 111 (2.019 Å). For antioxidant targets, chlorogenic acid targeted the binding pockets of Nrf2 by forming hydrogen bonds with Arg 517 (2.990 Å), Asp 21 (2.539, 2.457 Å), and Gly 10 (2.630 Å), and Keap1 via Arg 483 (3.271, 3.168, 2.696 Å), Ser 508 (2.712 Å), Gln 530 (3.131 Å), and Ser 555 (2.671 Å). Citric acid exhibited affinity for Nrf2 through Arg 502 (3.179 Å) and for Keap1 via multiple interactions with Ser 363 (3.200, 3.219, 3.200 Å). These findings suggest that the major compounds of *P. mume* effectively modulate both apoptotic and antioxidant signaling pathways through stable molecular interactions with their respective regulatory proteins. It should be noted that the relatively lower complex pLDDT score for Nrf2 (0.42) is attributable to the intrinsically disordered regions typical of transcription factor activation domains, whereas the high ipTM scores and localized hydrogen-bonding networks still support reliable local binding interactions within the active binding pocket.

## 3. Discussion

The clinical utility of conventional chemotherapeutic agents, such as cisplatin, is heavily restricted by dose-limiting systemic toxicities, therapeutic resistance, and secondary inflammatory damage to non-cancerous tissues [11,25]. Consequently, identifying multi-target phytotherapeutic agents that selectively sensitize cancer cells to chemotherapy while simultaneously safeguarding host normal cells and immune homeostasis represents a pivotal goal in oncology [12,26]. In the present study, we demonstrate that PME functions as an exceptional therapeutic adjuvant. It exerts a dual action: (1) cooperatively enhancing cisplatin-induced apoptosis in BRAF-mutated A375P human melanoma cells, and (2) exerting cytoprotective and anti-inflammatory effects in HaCaT and PBMCs by counteracting cisplatin-induced hyper-inflammatory stress. A key outcome of this investigation is the significant selective cytotoxicity exhibited by PME. In A375P melanoma cells, sub-toxic cisplatin (20 μM) combined with low-dose PME (0.1 μg/mL) markedly enhanced cancer cell death through mitochondrial-mediated intrinsic apoptosis, characterized by loss of mitochondrial membrane potential (Figure 3b,c), clear genomic DNA fragmentation (Figure 3a), and an elevated *Bax*/*Bcl-2* ratio coupled with *Caspase-3*/*8* activation (Figure 4). In our combination cytotoxicity analysis (Figure 2a), the observed cell death rate of 13.2% closely aligned with the Bliss independence additive expectation (13.4%), demonstrating an additive chemosensitizing effect rather than profound hyper-synergism. Importantly, the primary rationale for utilizing this low concentration window was to avoid off-target cytotoxicity against normal cells—as evidenced by preserved HaCaT viability (>100%, Figure 2b)—while effectively priming melanoma cells to chemotherapeutic stress. Mitochondria act as critical integration centers for cell death decisions. Disruption of membrane potential leads to outer membrane permeabilization and subsequent caspase cascades. Although a reduction in *Caspase-9* mRNA levels was observed following combination treatment (Figure 4c), this transient attenuation likely reflects late-stage transcriptional feedback regulation or selective transcript turnover kinetics during sustained drug exposure, rather than a failure of apoptotic initiation. Crucially, while this apoptotic machinery was vigorously engaged in cancer cells, identical treatment regimens preserved HaCaT viability (Figure 1c and Figure 2b). The chemosensitizing and cytoprotective properties of PME are closely linked to its rich phytochemical profile identified via LC-MS/MS (Supplementary Table S1). Beyond pure compounds, crude botanical extracts provide a distinct therapeutic advantage via multi-component synergy and the botanical entourage effect. Among the detected components, chlorogenic acid, caffeic acid, 4-*O*-caffeoylquinic acid, rutin, and quercetin have been extensively shown to overcome chemotherapeutic resistance by disrupting mitochondrial bioenergetics, generating targeted reactive oxygen species (ROS), and downregulating survival pathways. The recent literature highlights that natural polyphenols act as potent chemosensitizers by coordinating intrinsic apoptotic cascades and modulating drug sensitivity in refractory cancers [27,28,29,30]. Furthermore, low-molecular-weight organic acids, predominantly citric acid and aconitic acid, play crucial complementary roles by inducing metabolic stress in cancer cells while contributing to microenvironmental buffering and cytoprotection in normal tissues. Our molecular docking simulations (Figure 6 and Table 2) corroborate these findings, confirming that chlorogenic acid and citric acid establish stable hydrogen-bonding networks and favorable spatial affinities within the catalytic clefts of apoptosis regulators (*Caspase-3*, *Bcl-2*) and antioxidant defense proteins (Keap1/Nrf2). Beyond direct tumor cell killing, chemotherapy-induced toxicities are predominantly mediated by systemic inflammatory reactions and immune cell exhaustion [31]. Cisplatin is known to activate nuclear factor-κB (*NF-κB*) signaling, triggering a cytokine storm-like phenomenon in non-target immune populations, which contributes to off-target toxicities and organ injury [25,31]. Our results in primary PBMCs demonstrate that cisplatin exposure induced a profound inflammatory surge, marked by the overactivation of *NF-κB* and widespread upregulation of pro-inflammatory cytokine mRNAs (*IFN-γ*, *TNF-α*, *IL-2*, and *IL-10*) as well as cytotoxic effectors (*Granzyme B*, *Perforin*) and immune checkpoints (*PD-L1*, *CD80*). Such uncontrolled immune activation leads to autotoxicity and lymphopenia, severely impairing the host’s long-term anti-tumor immunity. In addition, co-treatment with PME efficiently attenuated this hyper-inflammatory response. By dampening cisplatin-induced *NF-κB* upregulation, PME restored PBMC viability to baseline levels (Figure 5a) and normalized cytokine expression patterns. The suppression of *NF-κB* pathways by natural bioactive agents is a well-established mechanism for mitigating chemotherapy-driven toxicities [26]. The downregulation of *Granzyme B* and *Perforin* mRNA transcripts back to physiological levels indicates the prevention of immune cell self-destruction. Furthermore, the reduction in cisplatin-induced *PD-L1* expression in PBMCs suggests that PME prevents chemotherapy-driven immune suppression and exhaustion [24]. Importantly, this attenuation of effector molecules should be interpreted with nuance: rather than indicating an impairment of host antitumor immunity, PME serves as an immunomodulatory buffer that prevents cisplatin-induced hyper-activation and inflammatory damage, thereby preserving viable immune homeostasis. Despite these promising findings, several limitations of this study should be noted. First, the apoptotic signaling and cytokine evaluations were predominantly conducted at the transcriptional (mRNA) level; future studies incorporating direct protein expression and cleavage kinetics (e.g., Western blot and ELISA) will be necessary to comprehensively validate the downstream execution. Second, while our in vitro investigations using A375P melanoma cells, HaCaT keratinocytes, and human primary PBMCs provide valuable mechanistic insights, further in vivo preclinical xenograft studies are warranted to verify systemic bioavailability, pharmacokinetics, and therapeutic synergy in complex physiological environments.

## 4. Materials and Methods

### 4.1. Chemicals and Reagents

All chemicals and reagents used in this study were of analytical grade and purchased from commercial suppliers. Dulbecco’s Modified Eagle Medium (DMEM), RPMI 1640, Fetal Bovine Serum (FBS), and Antibiotic–Antimycotic were purchased from Gibco (Grand Island, NY, USA). Crystal violet and cis-diamminedichloroplatinum(II) (Cisplatin) were purchased from Sigma-Aldrich (St. Louis, MO, USA), while the XTT Cell Viability Assay Kit was obtained from Biotium (Hayward, CA, USA). Genomic DNA and total RNA were isolated using kits from Qiagen (Hilden, Germany) and Invirustech (Gwangju, Republic of Korea) respectively. The JC-1 Mitochondrial Membrane Potential Assay Kit was from Invitrogen (Carlsbad, CA, USA). All primers for RT-qPCR were synthesized by Bioneer (Daejeon, Republic of Korea), and the Verso 1-step RT-qPCR Kit with SYBR Green and low ROX was purchased from Thermo Fisher Scientific (Waltham, MA, USA). *P. mume* fruits were purchased from a commercial farm in Gwangyang-si, Jeonnam-Gwangju Special Metropolitan City, Republic of Korea. The plant material was authenticated based on morphological and phytochemical characteristics, and a reference voucher specimen (Batch No. JN-PME-2025-01) was deposited at the Jeonnam Institute of Health and Environment.

### 4.2. LC-MS/MS Qualitative Analysis

Qualitative profiling of PME- and SPE-fractionated samples was performed using an HPLC system (LC-40, Shimadzu, Kyoto, Japan) coupled with a Q-TOF mass spectrometer (ZenoTOF 7600+, SCIEX, Marlborough, MA, USA). Chromatographic separation was achieved on analytical C18 columns (Capcell Pak C18 Aq, 150 × 2.0 mm, 3 μm, Osaka Soda Co., Osaka, Japan; Kinetex XB-C18, 100 × 2.0 mm, 2.6 μm, Phenomenex, Torrance, CA, USA) maintained at a constant flow rate of 0.2 mL/min. The mobile phases consisted of 0.1% formic acid in distilled water (Mobile Phase A) and 0.1% formic acid in acetonitrile (Mobile Phase B). The gradient elution profile was programmed as follows: 0–2 min, 0% B; 2–5 min, 0–5% B; 5–7 min, 5–15% B; 7–10 min, 15–30% B; 10–13 min, 30–50% B; 13–17 min, 50–100% B; 17–20 min, 100% B (isocratic); 20–20.1 min, re-equilibration to 0% B; and 20.1–30 min, 0% B. Mass spectrometry was operated in Information-Dependent Acquisition (IDA) scan mode in both positive and negative polarities with Zeno Pulsing enabled. Full-scan TOF-MS data were acquired over an *m*/*z* range of 50–1250, and TOF-MS/MS fragmentations were recorded over an *m*/*z* range of 20–750. Compound identification was performed by matching accurate precursor mass (*m*/*z*), retention times (RT), and MS/MS fragmentation patterns against High-Resolution spectral libraries (NIST) using Smart Confirmation algorithms.

### 4.3. Preparation of P. mume Ethanol Extracts

*P. mume* fruits were purchased from Gwangyang-si, Jeonnam-Gwangju Special Metropolitan City, Republic of Korea. The plant material was botanically authenticated based on morphological and phytochemical characteristics by our institutional researchers, and a reference voucher specimen (Batch No. JN-PME-2025-01) was deposited at the Jeonnam Institute of Health and Environment. The seeds were removed from the fresh fruits, and the deseeded pulp was immediately lyophilized. To evaluate the extraction efficiency across different solvent conditions, the dried *P. mume* powder was extracted using various concentrations of ethanol (60%, 80%, and 100%, *v*/*v*) at a solid-to-solvent ratio of 1:10 (*w*/*v*; 100 g of dried powder in 1000 mL of solvent). The extraction was performed by stirring and sonication under dark conditions at 60 °C for 3 h. Following extraction, each mixture was filtered, and the filtrate was concentrated using a rotary vacuum evaporator to remove ethanol. The remaining aqueous fraction was subsequently lyophilized to yield final powdered extracts (extraction yield: 16.7% *w*/*w*, dry weight basis). The dried extracts were stored at −80 °C until further use. For in vitro cell treatment, the extracts were dissolved in dimethyl sulfoxide (DMSO), ensuring that the final DMSO concentration in the culture medium did not exceed 0.1% (*v*/*v*).

### 4.4. Cell Culture

The human malignant melanoma cell line A375P was obtained from the Korean Cell Line Bank (Seoul, Republic of Korea). The normal human keratinocyte cell line HaCaT and PBMCs were obtained from Komabiotech (Seoul, Republic of Korea). A375P and HaCaT cells were maintained in DMEM, while PBMCs were cultured in RPMI-1640, both supplemented with 10% FBS and 1% Antibiotic–Antimycotic. All cells were incubated at 37 °C in a humidified 5% CO_2_ atmosphere.

### 4.5. Cytotoxicity and Combination Treatment Assay

A375P and HaCaT cells were seeded at a density of 1 × 10^4^ cells/well in 96-well plates. After 24 h of stabilization, the cells were treated with either PME (0–200 μg/mL) or cisplatin (0.01–100 μM) for 24 h to determine individual dose–response profiles. For combination assays, A375P cells were co-treated with a fixed sub-toxic dose of cisplatin and varying concentrations of the PME. Cell viability was evaluated using the crystal violet staining protocols. Briefly, cells were fixed with ice-cold methanol and stained with 1% crystal violet solution. After washing and drying, the retained dye was solubilized, and absorbance was measured at 570 nm using a microplate reader (Tecan Infinite M200 PRO, Männedorf, Switzerland). Cell viability was calculated using the following equation:

Cell viability (%) = (ODT/ODC) × 100%

where ODT and ODC represent the average optical density values of the treated experimental group and the untreated control group at 570 nm, respectively.

Conversely, the cell viability and metabolic activity of non-adherent PBMCs were evaluated using the XTT cell viability assay kit. Briefly, PBMCs were seeded at a density of 1 × 10^6^ cells/ well and treated with either PME (0.1 μg/mL), cisplatin (20 μM), or their combination for 16 h under specified conditions, after which the XTT working solution was added to each well according to the manufacturer’s instructions. Following incubation at 37 °C for 2 h, the absorbance of the formed water-soluble formazan dye was measured at 450 nm (with a reference wavelength of 660 nm) using a microplate reader (Tecan Infinite M200 PRO, Männedorf, Switzerland). Cell viability was determined using the same calculation method as described in the equation. The half-maximal inhibitory concentration (IC_50_) values and dose–response curves were calculated and generated using GraphPad Prism 6.0 (GraphPad Software, San Diego, CA, USA) by fitting a non-linear regression model.

### 4.6. DNA Fragmentation

A375P cells were seeded at a density of 1 × 10^5^ cells/flask in T-25 culture flasks. After 24 h of stabilization, the cells were treated with either PME (0.1 μg/mL), cisplatin (20 μM), or their combination for 24 h. Subsequently, genomic DNA was extracted using the QIAamp DNA Blood Mini Kit (Qiagen, Hilden, Germany) according to the manufacturer’s protocol. Equal amounts of purified genomic DNA (5 μg) were separated by electrophoresis on a 1.5% agarose gel containing SYBR Safe DNA Gel Stain (Invitrogen, Carlsbad, CA, USA) at 100 V. DNA fragments were visualized and photographed under ultraviolet (UV) illumination using a gel documentation system.

### 4.7. Mitochondrial Membrane Potential

Mitochondrial membrane potential was evaluated using a MitoProbeTM JC-1 Assay Kit (Invitrogen, Carlsbad, CA, USA) according to the manufacturer’s instructions. Cells were seeded onto SPL 8-well cell culture slides (2 × 10^4^ cells/well) for qualitative fluorescence microscopy analysis or black 96-well plates (1 × 10^4^ cells/well) for quantitative microplate reader analysis. Following stabilization, cells were treated with the cisplatin, PME, or their combination for 16 h. For the positive control, cells were treated with 10 μM CCCP for 15 min prior to JC-1 staining. Cells were then stained with 1× JC-1 working solution for 15 min at 37 °C in the dark and washed with 1× Assay Buffer. For qualitative assessment, mitochondrial depolarization was visualized using a fluorescence microscope (Olympus BX53, Tokyo, Japan) equipped with cellSens Standard software (version 1.18, Olympus) by assessing the shift from red J-aggregates to green monomers. For quantitative analysis, fluorescence intensities were measured using a microplate reader (Tecan Infinite M200 PRO, Männedorf, Switzerland) at excitation/emission wavelengths of 488/527 nm (green monomers) and 488/590 nm (red aggregates). The relative mitochondrial membrane potential was determined as the ratio of red to green fluorescence intensity (F590/F527).

### 4.8. Gene Expression Analysis (One Step RT-qPCR)

Total RNA was extracted from treated A375P cells or PBMCs using an RNA isolation kit (Invirustech, Gwangju, Republic of Korea) according to the manufacturer’s protocol. Briefly, cells were seeded into 24-well cell culture plates (SPL Life Sciences, Pocheon, Republic of Korea) at a density of 5 × 10^4^ cells/well and allowed to stabilize. Following stabilization, cells were treated with cisplatin, PME, or their combination for 16 h (adherent and floating fractions) or 30 h (total cells). The concentration and purity of the extracted RNA were determined by measuring the absorbance at 260 and 280 nm. To quantify mRNA expression levels, one-step real-time RT-qPCR was performed on an Applied Biosystems StepOnePlus^TM^ system (Thermo Fisher Scientific, Waltham, MA, USA). In this procedure, reverse transcription and cDNA amplification were carried out sequentially in a single reaction tube using a Verso 1-step RT-qPCR Kit (SYBR Green with low ROX). Briefly, 100 ng of total RNA was added to the reaction mixture containing specific primers and the one-step master mix. The thermal cycling conditions consisted of an initial reverse transcription step at 50 °C for 15 min and 95 °C for 15 min, followed by PCR activation and 40 cycles of denaturation, annealing, and extension. Target genes included apoptotic markers (*Bax*, *Bcl-2*, and Caspases), pro-inflammatory and Th1 cytokines (*IFN-γ*, *TNF-α*, and *IL-2*), anti-inflammatory and Th2 cytokines (*IL-4* and *IL-10*), an immunoregulatory cytokine (*IL-12*), cytotoxic effector molecules (*Granzyme B* and *Perforin*), and inflammatory signaling pathways and immune checkpoints (*NF-κB*, *PD-L1*, and *CD80*). The specific primer sequences used for these genes are summarized in Table 1. Data were normalized to *GAPDH* and analyzed via the 2^−ΔΔCt^ method.

### 4.9. In Silico Molecular Docking via Boltz-2

To elucidate the interactions between bioactive compounds identified via LC-MS/MS and potential apoptotic or cytoprotective targets—including *Caspase-3* (PDB ID: 1GFW), *Bcl-2* (PDB ID: 2O21), Nrf2 (PDB ID: 2LZ1), and Keap1 (PDB ID: 4XMB)—molecular docking was performed using Boltz-2, an open-source biomolecular interaction prediction framework inspired by the Boltz-2 architecture, hosted on the Neurosnap platform. Ligand SMILES strings for chlorogenic acid and citric acid were retrieved from PubChem. Structural reliability and binding stability within the active pockets were evaluated using complex pLDDT and ipTM metrics.

### 4.10. Statistical Analysis

All data are presented as the mean ± SEM of at least three independent experiments. Statistical significance was assessed using one-way or two-way ANOVA followed by Tukey’s multiple comparisons test in GraphPad Prism 5.0. A *p*-value < 0.05 was considered statistically significant.

## 5. Conclusions

In conclusion, this study demonstrates that PME functions as an effective multi-target chemotherapeutic adjuvant in melanoma therapy. The primary novel contributions of this work include:

1. Selective Chemosensitization: Standardized PME cooperates with sub-toxic doses of cisplatin to promote selective apoptotic cell death in BRAF-mutated A375P melanoma cells via mitochondrial depolarization and *Bax*/*Bcl-2* rebalancing, while maintaining cytocompatibility in normal HaCaT keratinocytes.
2. Immune Homeostasis Modulation: In primary human PBMCs, PME mitigates cisplatin-induced hyper-inflammatory cytokine surges and downregulates over-activated cytotoxic effectors (*Granzyme B*, *Perforin*) and checkpoint molecules (*PD-L1*) through *NF-κB* suppression, preventing chemo-induced immune autotoxicity.
3. Phytochemical & Structural Rationale: High-resolution LC-MS/MS and Boltz-2 molecular modeling reveal that the dual anticancer and cytoprotective bioactivities of PME stem from the complementary interactions of major polyphenols (chlorogenic acid) and organic acids (citric acid) with pivotal apoptotic and antioxidant regulators. Collectively, these findings highlight the potential of PME as a complementary candidate to improve therapeutic outcomes and alleviate off-target toxicities in cisplatin-based melanoma regimens.

## Figures and Tables

**Figure 1:**
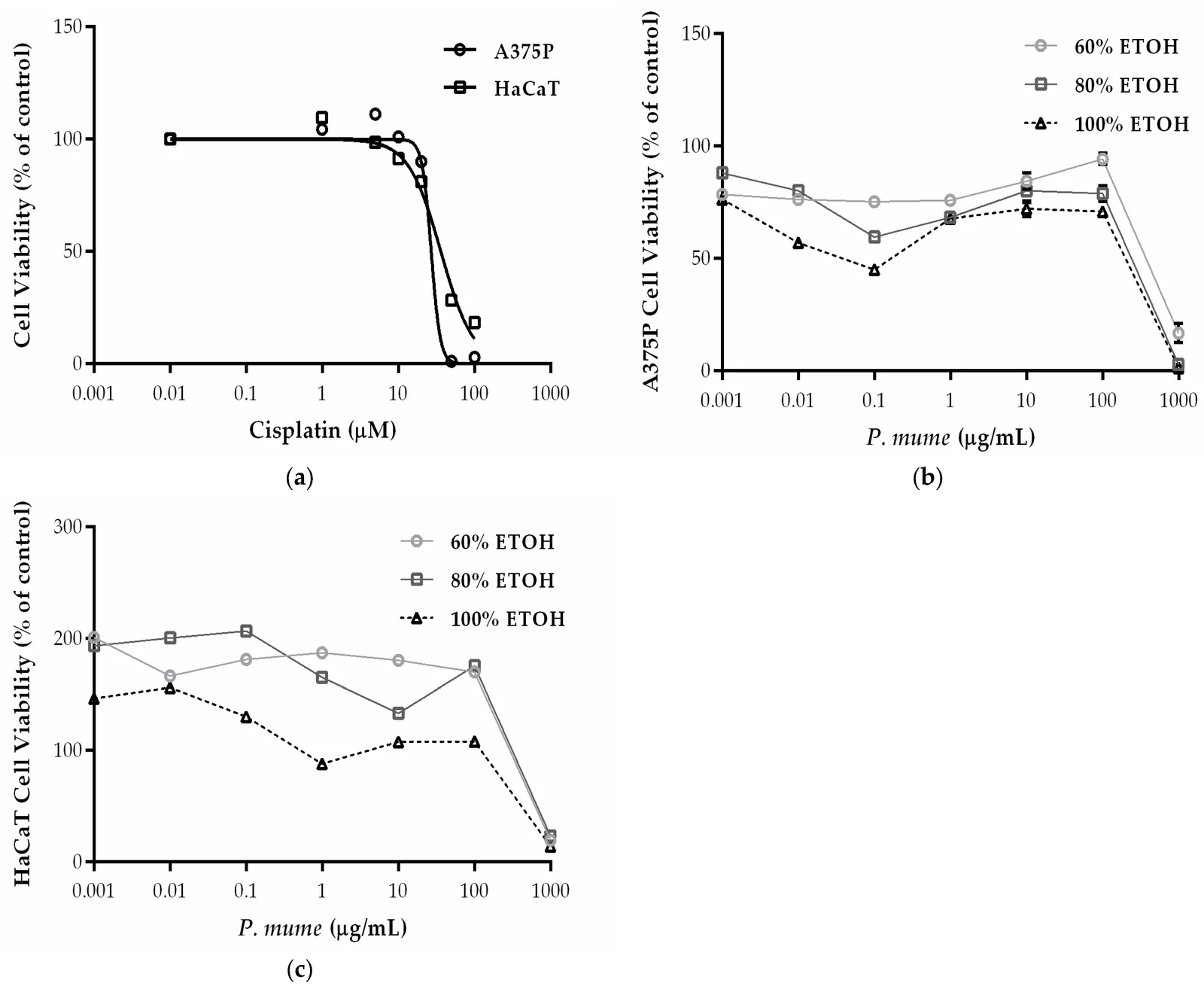
Establishment of cisplatin dosage and selective growth inhibitory effects of PME on A375P and HaCaT cells. (**a**) Dose–response curves of cisplatin. To determine the optimal sub-toxic concentration for combination therapy, A375P and HaCaT cells were treated with cisplatin (0.01–100 μM). Based on the growth inhibition profiles, 20 μM of cisplatin was selected for further synergistic studies. (**b**) Growth inhibition in A375P cells. Cells were treated with 60%, 80%, and 100% EtOH extracts of *P. mume* (0.001–1000 μg/mL). All extracts exhibited dose-dependent inhibitory effects on cancer cell proliferation. (**c**) Cytocompatibility in HaCaT cells. Normal keratinocytes showed stable or increased viability at lower concentrations (<100 μg/mL), while a significant drop in viability was observed at 1000 μg/mL across all groups due to the acidic pH of the extract. Data are expressed as the mean ± standard error of the mean (SEM) of three independent experiments (*n* = 3). Error bars smaller than the symbols are not shown.

**Figure 2:**
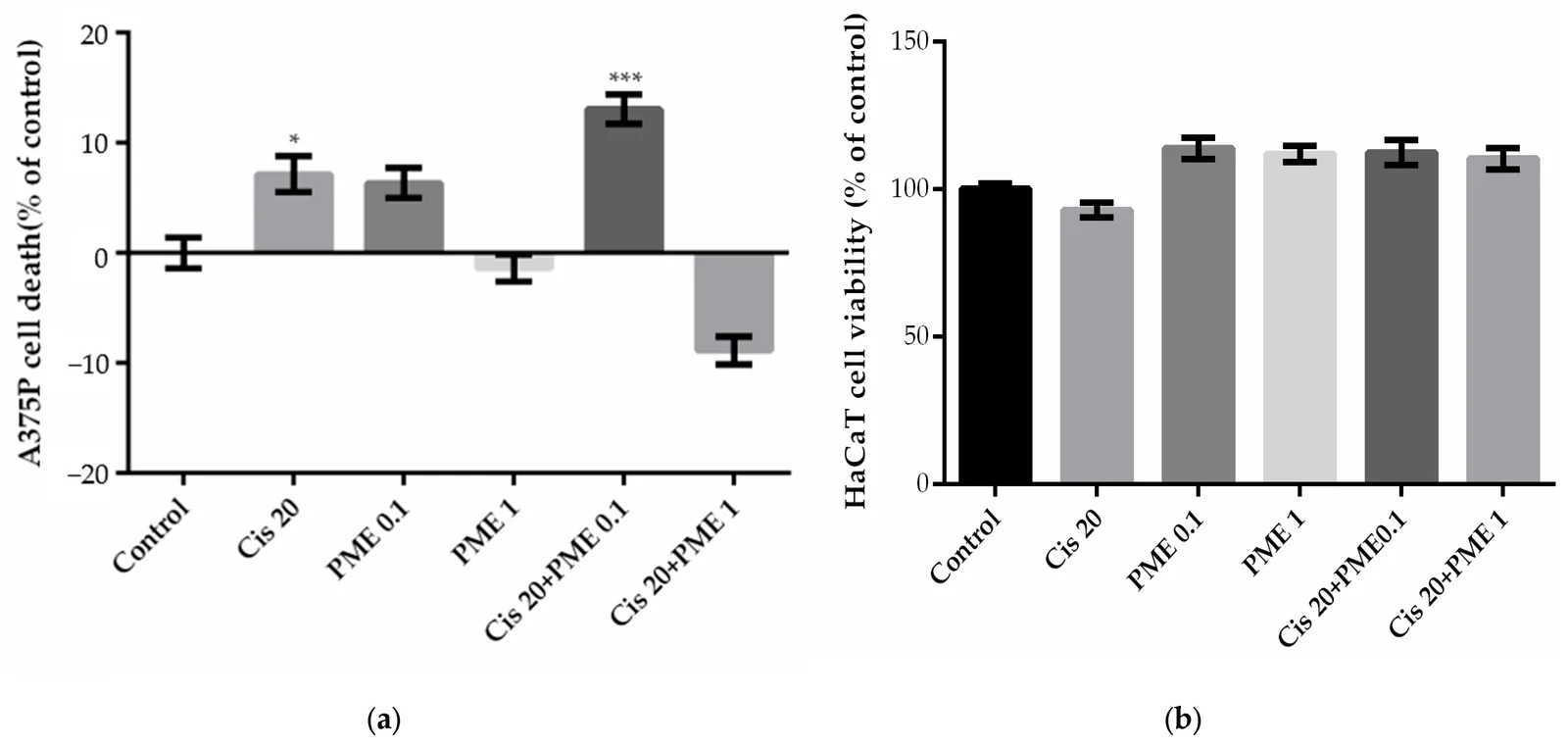
Synergistic anticancer activity and selective cytoprotective effects of PME in combination with cisplatin. (**a**) Differential modulation of cell death in A375P melanoma cells. Cells were co-treated with cisplatin (20 μM) and PME (0.1, 1 μg/mL) for 16 h. Low-dose PME (0.1 μg/mL) cooperatively enhanced cisplatin-induced cell death (13.2%, *** *p* < 0.001). (**b**) Cell viability of HaCaT normal keratinocytes relative to untreated controls. PME effectively rescued HaCaT cells from cisplatin-induced cytotoxicity, maintaining viability > 100%. Data represent Mean ± SEM (*n* = 3). * *p* < 0.05 and *** *p* < 0.001 vs. Control.

**Figure 3:**
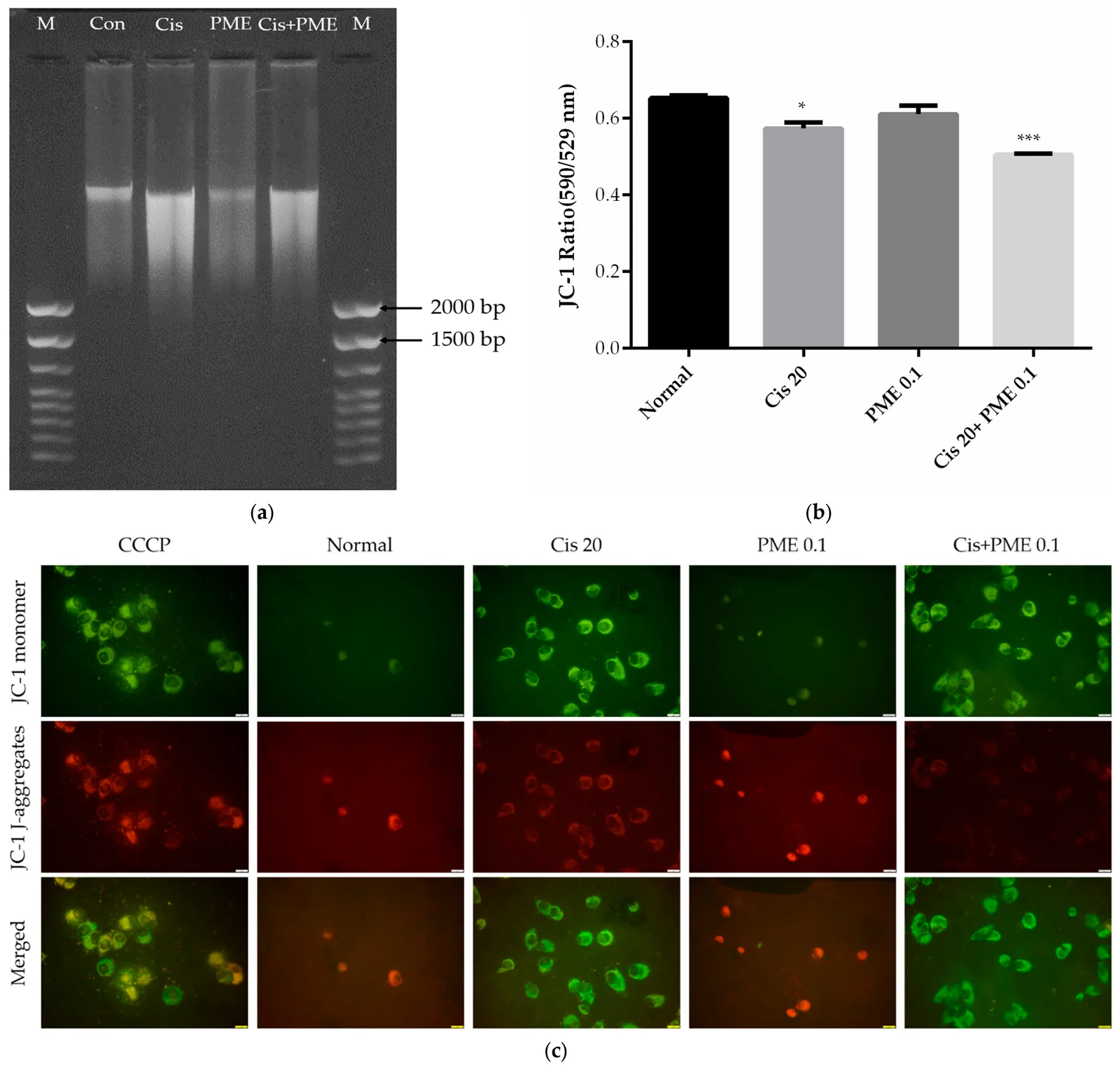
Induction of apoptotic DNA fragmentation and loss of mitochondrial membrane potential by combined treatment. (**a**) Representative agarose-gel electrophoresis showing DNA fragmentation in A375P cells (arrows indicate 2000 bp and 1500 bp DNA ladder size markers). (**b**) Quantitative analysis of the JC-1 fluorescence ratio (590/529 nm ratio), representing changes in mitochondrial membrane potential. (**c**) Representative fluorescence images of JC-1 staining in A375P cells. Green fluorescence indicates JC-1 monomers (depolarized membrane), red fluorescence indicates JC-1 J-aggregates (intact membrane), and orange indicates merged signals. CCCP (10 μM) was used as a positive control for mitochondrial membrane potential depolarization. Data in (**b**) are presented as mean ± SEM (*n* = 3). * *p* < 0.05 and *** *p* < 0.001 vs. Normal control. Original magnification: 400×, Scale bars = 20 μm.

**Figure 4:**
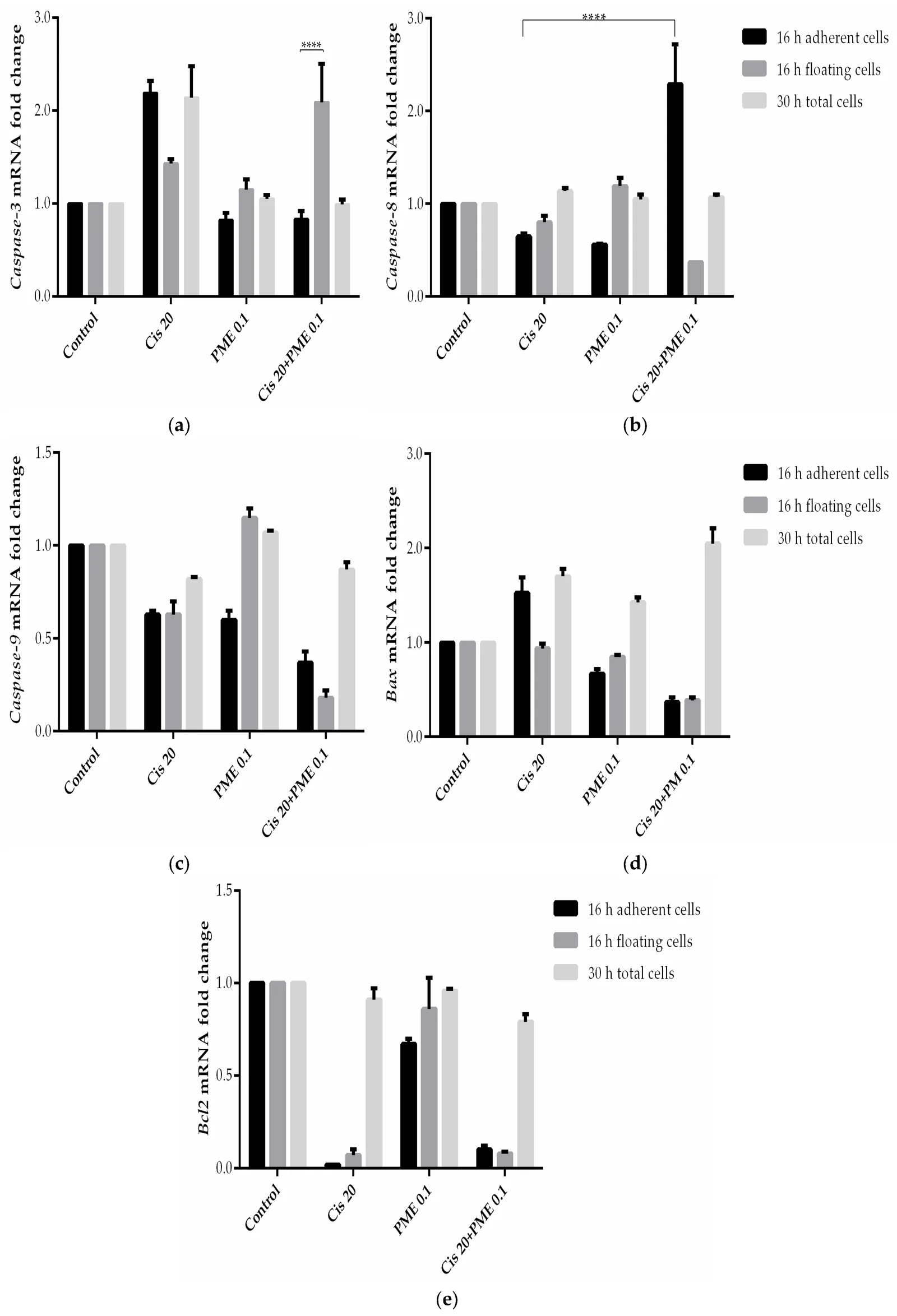
Modulation of apoptosis-related mRNA expression by co-treatment with cisplatin and PME in A375P melanoma cells. RT-qPCR analysis of apoptotic markers across three distinct cell fractions (16 h adherent cells, 16 h floating cells, and 30 h total cells): (**a**) *Caspase-3*, (**b**) *Caspase-8*, (**c**) *Caspase-9*, (**d**) *Bax*, and (**e**) *Bcl2* mRNA expression fold changes relative to untreated controls. Cells were treated with control, cisplatin (20 μM), PME (0.1 μg/mL), or their combination (Cis 20 μM + PME 0.1 μg/mL). mRNA expression levels were normalized to *GAPDH*. Data represent mean ± SEM (*n* = 3). **** *p* < 0.0001, indicated for key comparisons between specified groups as determined by two-way ANOVA followed by Tukey’s multiple comparisons test. Unmarked comparisons indicate no statistical significance (*p* > 0.05).

**Figure 5:**
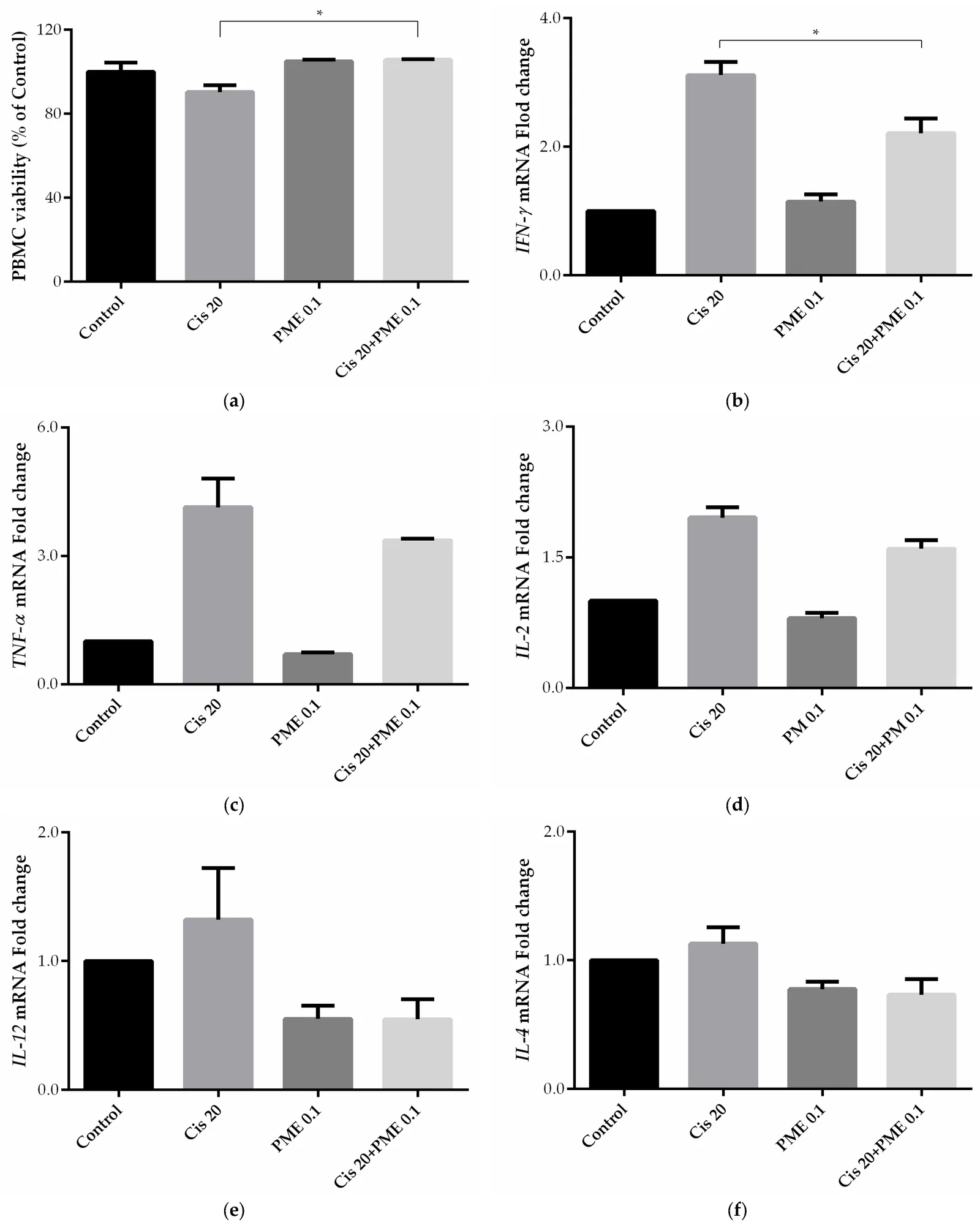

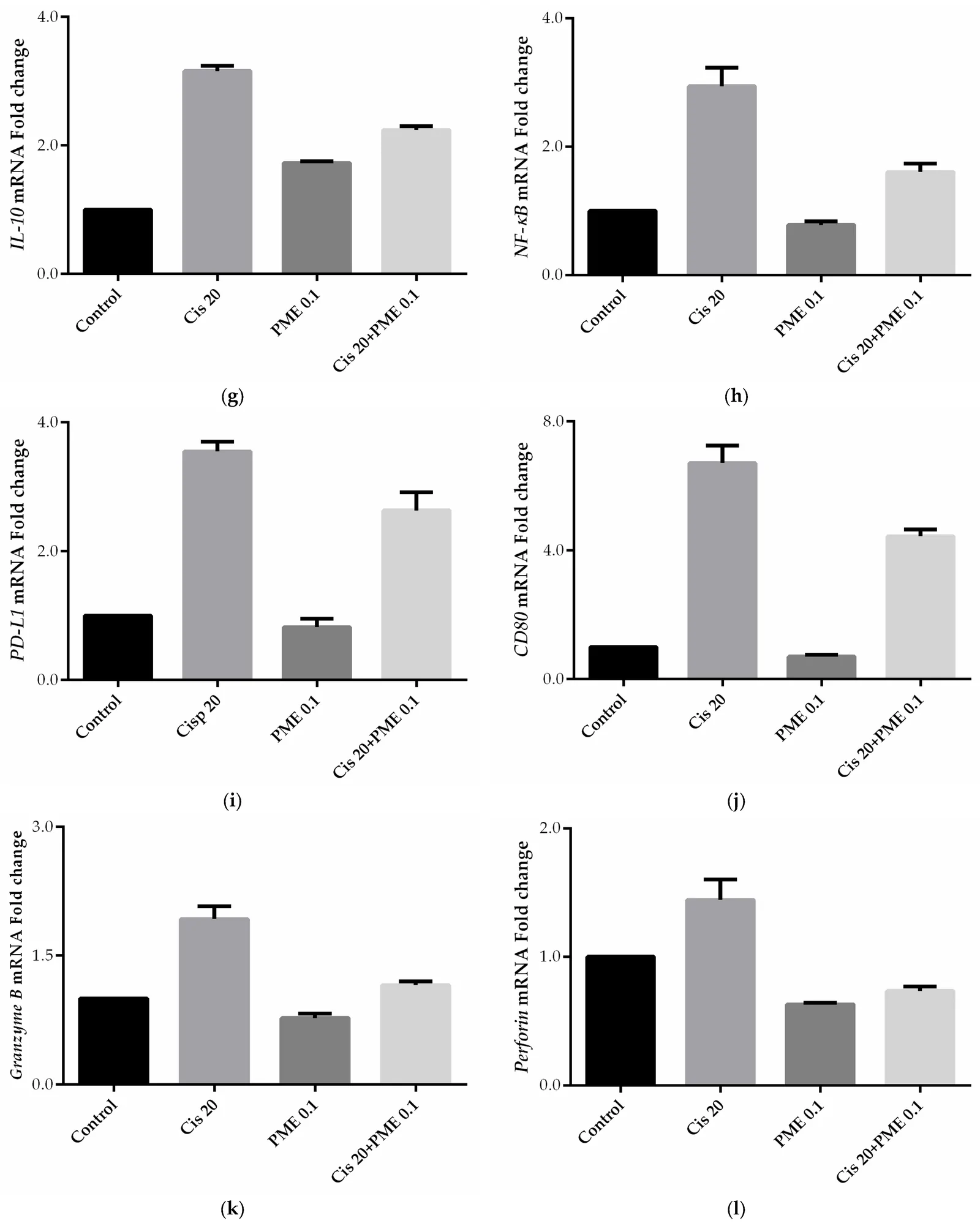
Protective effects of PME against cisplatin-induced cytotoxicity and inflammatory responses in PBMCs. Primary PBMCs were treated with cisplatin (20 μM), PME (0.1 μg/mL), or combination of both for 16 h. (**a**) PBMC viability was determined by cell cytotoxicity assay. (**b**–**l**) Relative mRNA expression levels of inflammatory cytokines, transcriptional factors, immune checkpoints, and cytotoxic effectors were measured by RT-qPCR: (**b**) *IFN-γ*, (**c**) *TNF-α*, (**d**) *IL-2*, (**e**) *IL-12*, (**f**) *IL-4*, (**g**) *IL-10*, (**h**) *NF-κB*, (**i**) *PD-L1*, (**j**) *CD80*, (**k**) *Granzyme B*, and (**l**) *Perforin* values represent fold changes normalized to *GAPDH* and expressed relative to the untreated control group (1.0). Data are presented as mean ± SEM (*n* = 3). * *p* < 0.05 versus the cisplatin-treated group.

**Figure 6:**
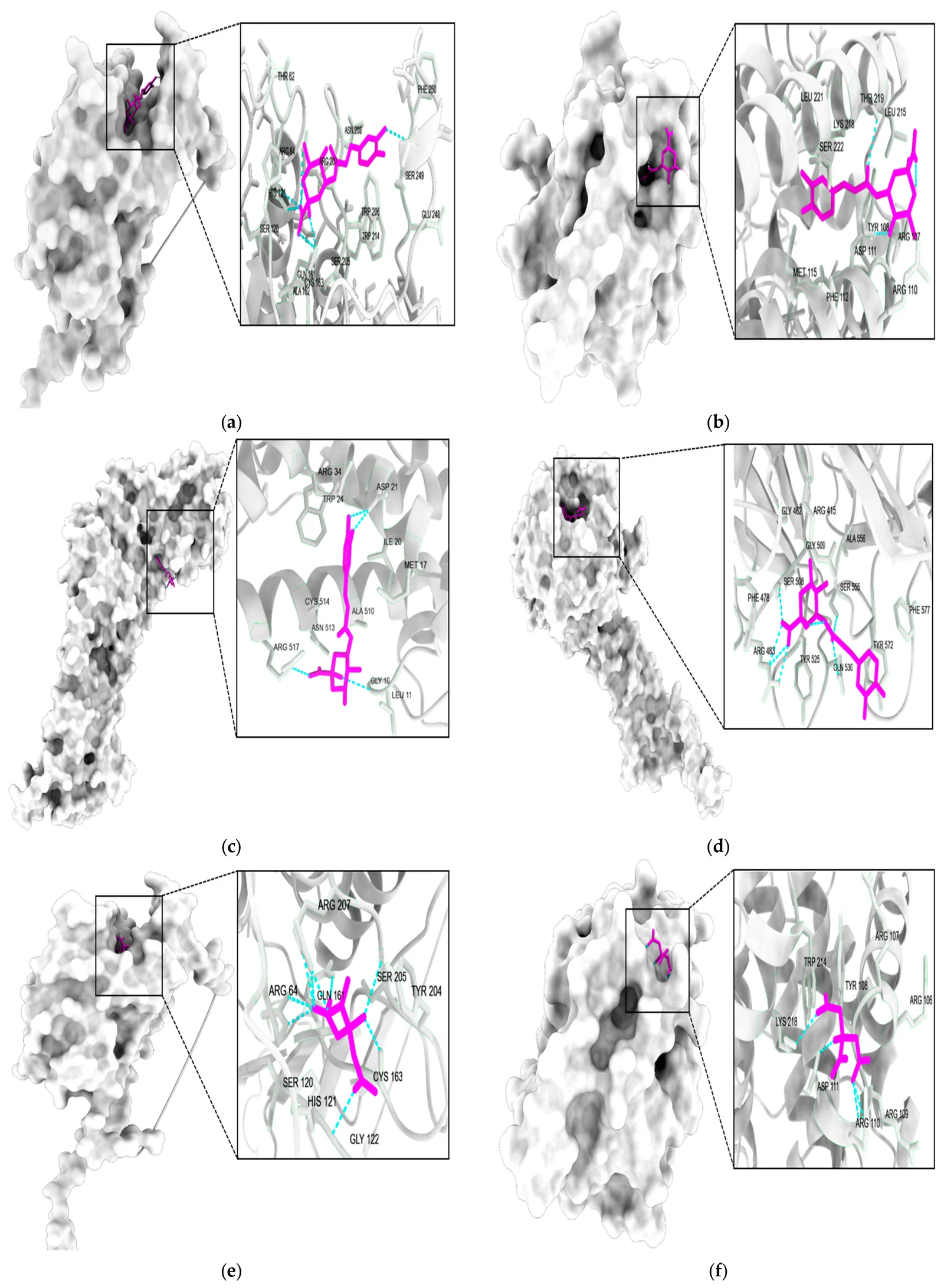

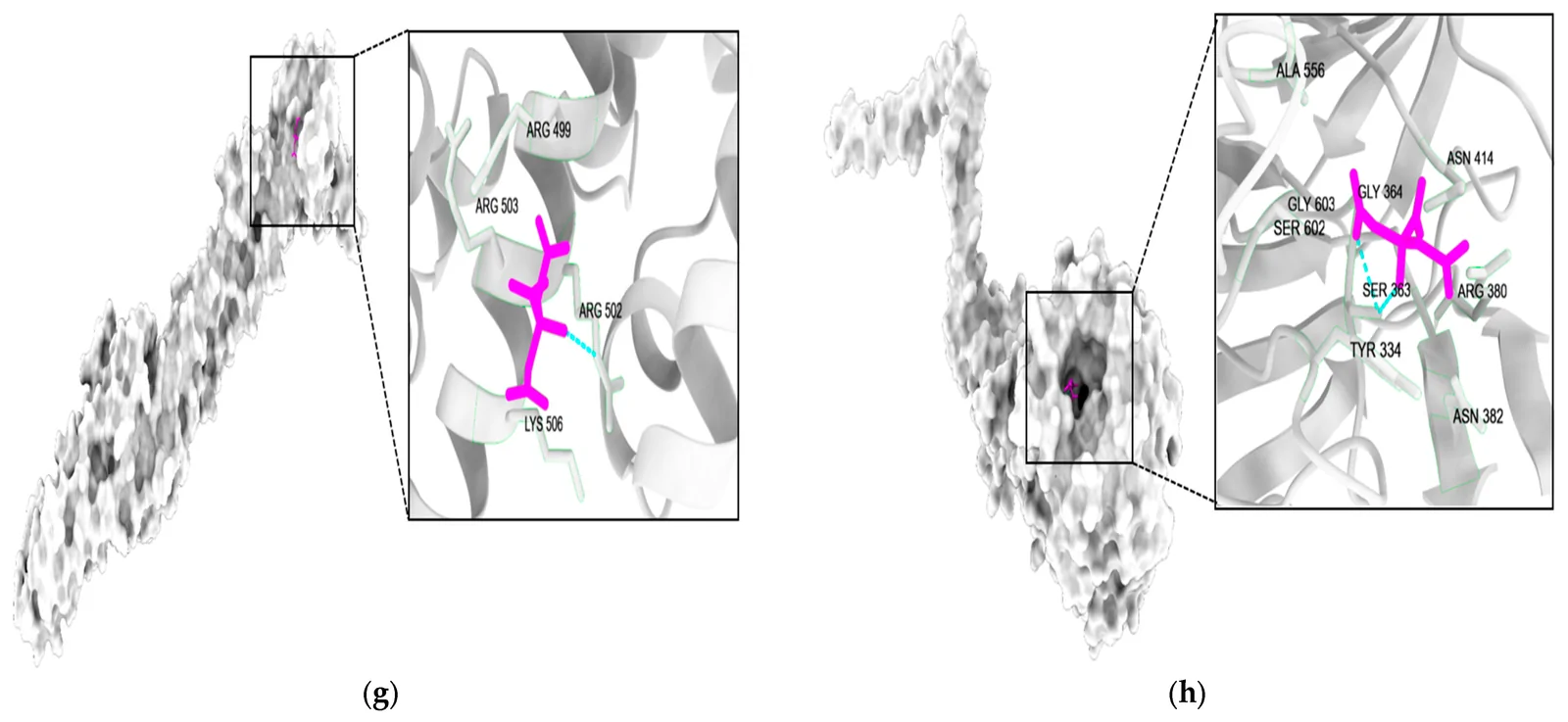
Comparative molecular docking analysis of chlorogenic acid and citric acid with key pro-apoptotic and antioxidant target proteins. The overall binding poses (left) and detailed molecular interactions (right) are shown for chlorogenic acid with (**a**) *Caspase-3* (PDB: 1GFW), (**b**) *Bcl-2* (PDB: 2O21), (**c**) Nrf2 (PDB: 2LZ1), and (**d**) Keap1 (PDB: 4XMB), and for citric acid with (**e**) *Caspase-3* (PDB: 1GFW), (**f**) *Bcl-2* (PDB: 2O21), (**g**) Nrf2 (PDB: 2LZ1), and (**h**) Keap1 (PDB: 4XMB). Cyan dashed lines indicate hydrogen bonding interactions with key amino acid residues, and ligands are highlighted in magenta.

**Table 1:** Primers used for RT-qPCR analysis.

Target Gene	Sequence (5′ → 3′)	Tm (°C)	Product Size (bp)	References
*GAPDH*	(F)ACAACTTTGGTATCGTGGAAG	55.2	100	[18]
	(R)CCATCACGCCACAGTTTC	55.5		
*Caspase-3*	(F)ATGGAAGCGAATCAATGGA	53.5	137	[19] (modified)
	(R)TGTACCAGACCGAGATGTC	54.9		
*Caspase-8*	(F)CTGCTGGGGATGGCC	55.1	113	In this study
	(R)TCGCCTCGAGGACATCG	54		
*Caspase-9*	(F)TGTCTACGGCACAGATGG	55.4	175	In this study
	(R)GGACTCGTCTTCAGGGG	54.8		
*Bax*	(F)TGGCAGCTGACATGTTTTC	55.5	195	[20]
	(R)TCACCCAACCACCCTG	54.1		
*Bcl-2*	(F)TCGCCCTGTGGATGAC	54.5	134	[21]
	(R)CAGAGACAGCCAGGAGAA	54.5		
*IFN-γ*	(F)TCCCATGGGTTGTGTGT	54.3	198	In this study
	(R)AAGCACCAGGCATGAAATC	55.2		
*TNF-α*	(F)CCCAGGGACCTCTCTCTAA	55.7	96	[22]
	(R)GTTTGCTACAACATGGGCTA	55.1		
*IL-2*	(F)TCAAACCTCTGGAGGAAGT	54.1	154	In this study
	(R)ATGGTTGCTGTCTCATCAG	53.9		
*IL-12*	(F)CATGGTGGATGCCGTTC	54.7	132	In this study
	(R)ACCTCCACCTGCCGA	55.1		
*IL-4*	(F)CCAACTGCTTCCCCCT	54.2	149	In this study
	(R)CTGTTACGGTCAACTCGG	54.2		
*IL-10*	(F)ACTTTAAGGGTTACCTGGGT	54.6	111	[23]
	(R)TCACATGCGCCTTGATG	54.2		
*NF-κB (p65)*	(F)ATGTGGAGATCATTGAGCAG	54.2	150	In this study
	(R)CTGGTCCTGTGTAGCCAT	54.9		
*PD-L1*	(F)TGCCGACTACAAGCGAA	55.1	83	[24] (modified)
	(R)TGACTGGATCCACAACCA	54.3		
*CD80*	(F)CTCACTTCTGTTCAGGTGTT	54.3	119	In this study
	(R)CTTTTGCCAGTAGATGCGA	54.7		
*Granzyme B*	(F)CTGGGAAAACACTCACACA	54.3	106	In this study
	(R)ACAACTCAATGGTACTGTCG	54.5		
*Perforin*	(F)CCTCTGTGAAAATGCCCTAC	55.2	164	In this study
	(R)AGTCGTTGCGGATGCTA	54.8		

**Table 2:** Molecular docking parameters and binding affinities of chlorogenic acid and citric acid with target proteins.

Target Protein (PDB ID)	Major Ligand	ipTM Score	Complex pLDDT
*Bcl-2* (2O21)	Chlorogenic acid	0.8804	0.7645
	Citric acid	0.6325	0.7274
*Caspase-3* (1GFW)	Chlorogenic acid	0.9472	0.9074
	Citric acid	0.8930	0.9253
Keap1 (4XMB)	Chlorogenic acid	0.9093	0.8642
	Citric acid	0.8639	0.877
Nrf2 (2LZ1)	Chlorogenic acid	0.8594	0.4239
	Citric acid	0.8244	0.4235

## Data Availability

The data presented in this study are available on request from the corresponding author.

## Supplementary Materials

The following supporting information can be downloaded at: https://www.mdpi.com/article/10.3390/molecules31203786/s1, Table S1: LC-MS/MS identification of bioactive compounds in *Prunus mume* extract.
