# Supplementary material for: Prunus mume Extract Cooperates with Cisplatin to Induce Melanoma Apoptosis and Regulate Immune Responses
Source: Molecules. 2026 Oct 21;31(20):3786. doi: 10.3390/molecules31203786 (PMC13707659; doi:10.3390/molecules31203786)

Chlorogenic acid and its Derivatives, Metabolites

Chlorogenic acid

Index	Sample Name	Component Name	Expected RT	Area	Adduct / Charge	Formula	Precursor Mass	Found At Mass	Mass Error (ppm)	Mass Error...	Isoto... Confi...	Library Confi...	Library Hit	Library Score	Isotope Ratio...	U...
1	BK_DW_1uL	Chlorgenic acid	16.08	N/A	[M+H]+	C16H18O9	355.1024	N/A	N/A					N/A	N/A	
2	Maesil_x100	Chlorgenic acid	16.08	4.101e5	[M+H]+	C16H18O9	355.1024	355.0993	-8.5				Chlorogenic acid (HR NIST) [Smart Confirmation]	100.0	3.0	

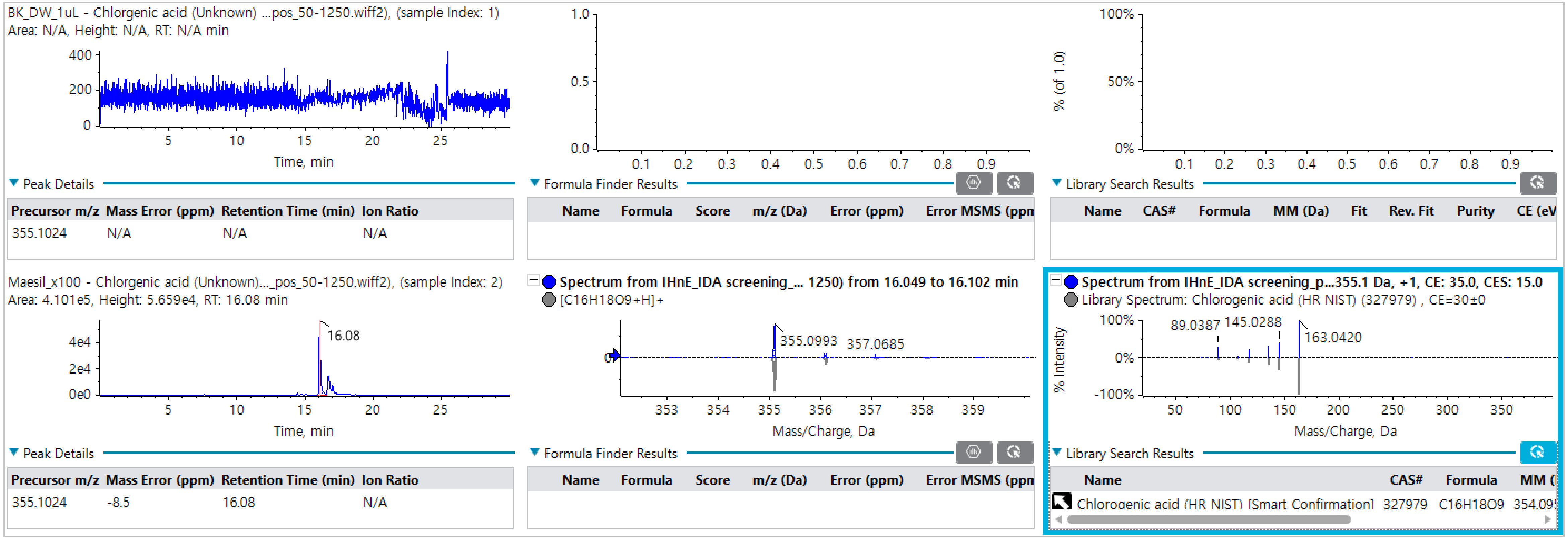

Chlorogenic acid and its Derivatives, Metabolites

Neochlorogenic acid

Index	Sample Name	Component Name	Expected RT	Area	Adduct / Charge	Formula	Precursor Mass	Found At Mass	Mass Error (ppm)	Mass Error...	Isoto... Confi...	Library Confi...	Library Hit	Library Score	Isotope Ratio...	U...
1	BK_DW_1uL	Neochlorogenic...	16.08	N/A	[M-H]-	C16H18O9	353.0878	N/A	N/A	■	■	■		N/A	N/A	☑
2	Maesil_x100	Neochlorogenic...	16.08	2.852e6	[M-H]-	C16H18O9	353.0878	353.0868	-2.9	✓	✓	✓	Neochlorogenic acid (HR NIST)	99.2	4.6	☑

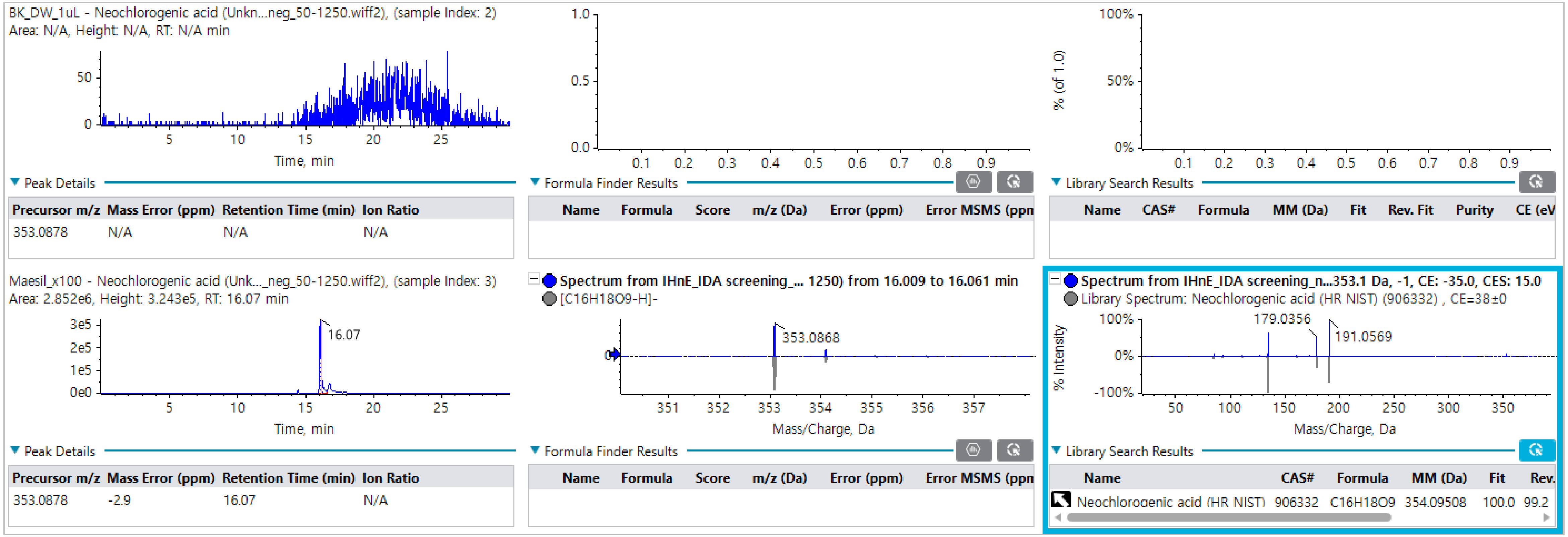

Chlorogenic acid and its Derivatives, Metabolites

Caffeic acid (M-H_2O)

	Index	Sample Name	Component Name	Expected RT	Area	Adduct / Charge	Formula	Precursor Mass	Found At Mass	Mass Error (ppm)	Mass Error...	Isoto... Confi...	Library Confi...	Library Hit	Library Score	Isotope Ratio...	U...
	2	BK_DW_1uL	Caffeic acid(-H2...	16.18	3.998e2	[M+H]+	C9H6O3	163.0390	163.0375	-9.2				No Acquired MSMS	N/A	73.8	
▸	4	Maesil_x100	Caffeic acid(-H2...	16.72	1.857e5	[M+H]+	C9H6O3	163.0390	163.0390	0.0	✓	✓	✓	Caffeic acid (HR NIST) [Smart Confirmation]	94.2	0.4	

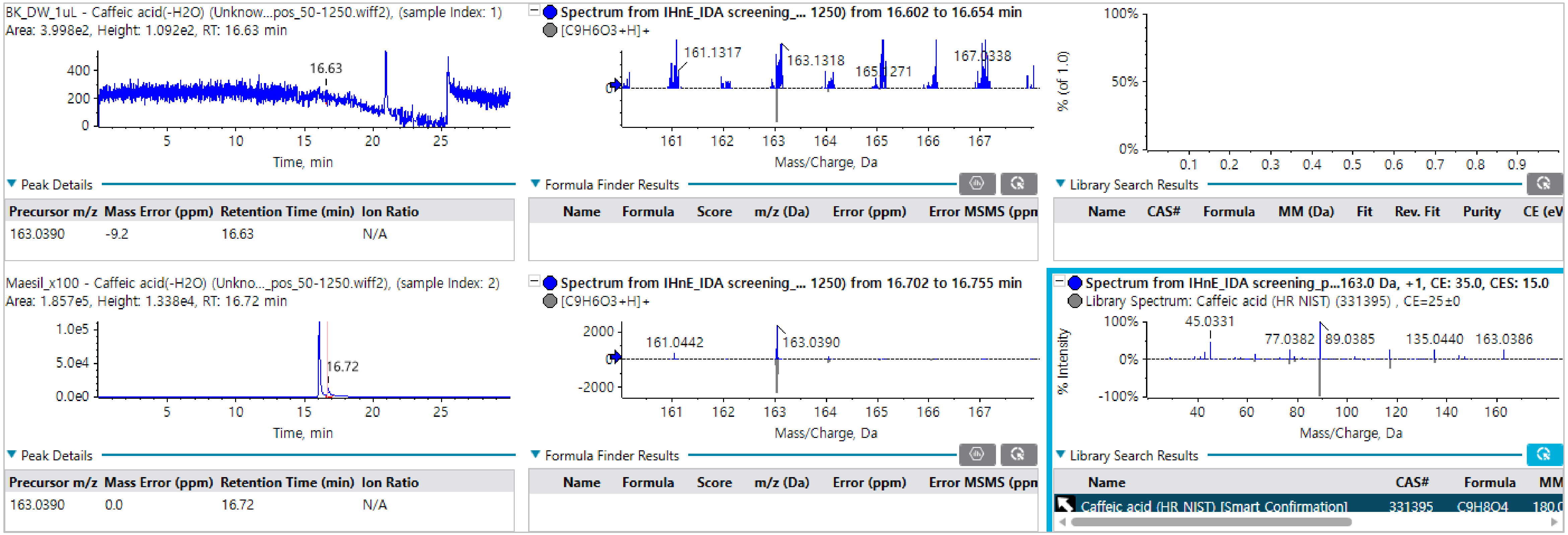

Chlorogenic acid and its Derivatives, Metabolites

4-O-Caffeoylquinic acid

Index	Sample Name	Component Name	Expected RT	Area	Adduct / Charge	Formula	Precursor Mass	Found At Mass	Mass Error (ppm)	Mass Error...	Isoto... Confi...	Library Confi...	Library Hit	Library Score	Isotope Ratio...	U...
3	BK_DW_1uL	4-O-caffeoylqui...	12.31	N/A	[M+Na]+	C16H18O9	377.0843	N/A	N/A	■	■	■		N/A	N/A	☑
6	Maesil_x100	4-O-caffeoylqui...	16.07	2.295e5	[M+Na]+	C16H18O9	377.0843	377.0843	0.0	✓	✓	✓	4-O-Caffeoylquinic acid (HR NIST)	98.8	3.7	☑

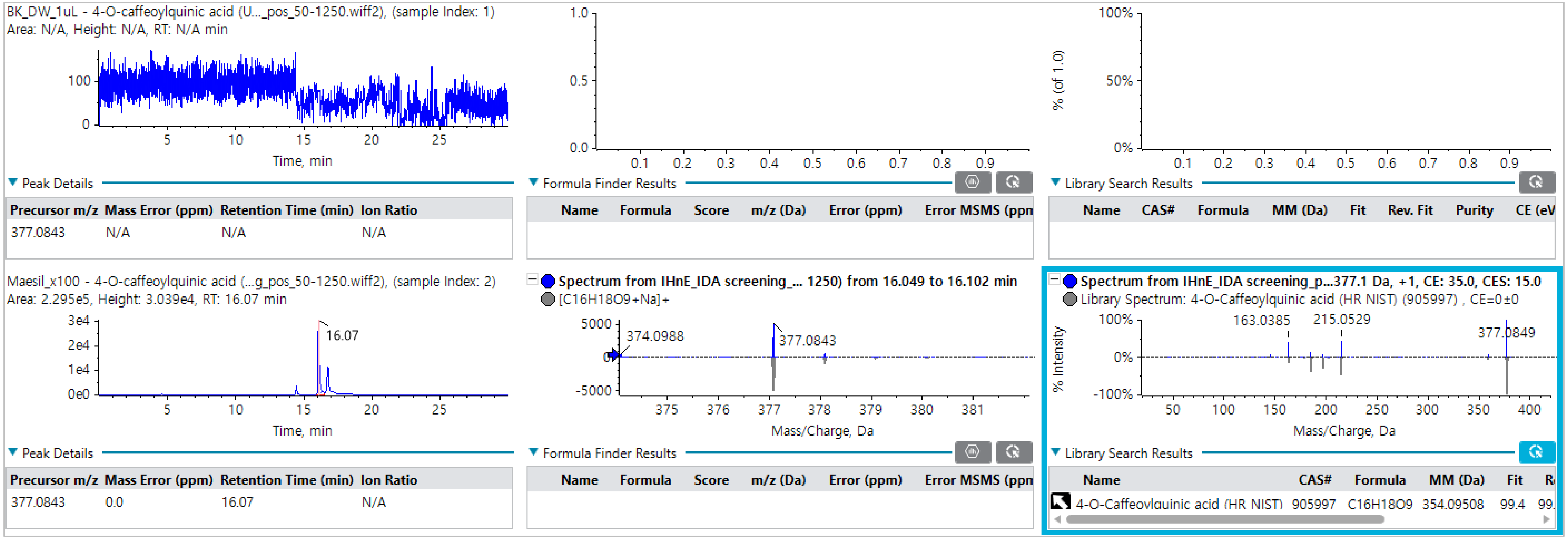

Citric acid and its Derivatives, Metabolites

Citric acid

	Index	Sample Name	Component Name	Expected RT	Area	Adduct / Charge	Formula	Precursor Mass	Found At Mass	Mass Error (ppm)	Mass Error...	Isoto... Confi...	Library Confi...	Library Hit	Library Score	Isotope Ratio...	U...
	4	BK_DW_1uL	Citric acid	14.41	2.633e3	[M+H]+	C6H8O7	193.0343	193.0337	-3.2	✓	▲	■	No Acquired MSMS	N/A	19.0	☑
▸	8	Maesil_x100	Citric acid	14.41	8.780e5	[M+H]+	C6H8O7	193.0343	193.0344	0.9	✓	✓	✓	Citric acid (HR NIST)	99.7	0.8	☑

BK_DW_1uL - Citric acid (Unknown) 193...._pos_50-1250.wiff2), (sample Index: 1)
Area: 2.633e3, Height: 4.241e2, RT: 14.43 min

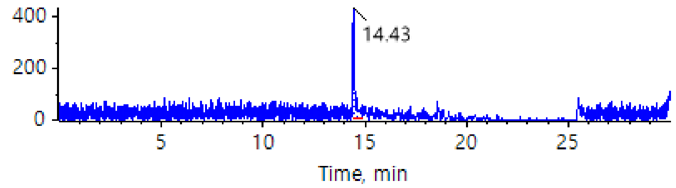

Spectrum from IHnE_IDA screening_... 1250) from 14.418 to 14.470 min
[C6H8O7+H]+

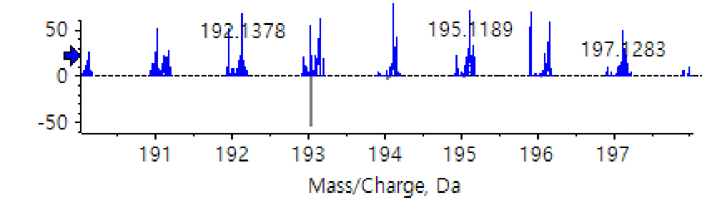

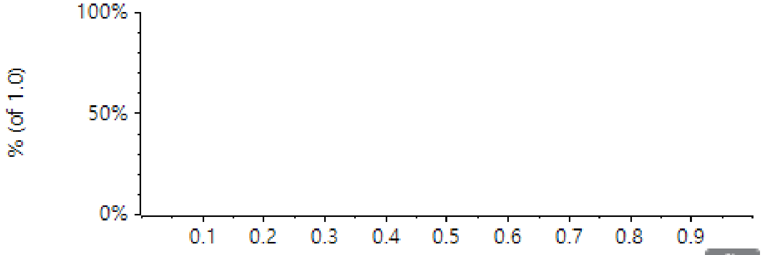

▼ Peak Details

Precursor m/z	Mass Error (ppm)	Retention Time (min)	Ion Ratio
193.0343	-3.2	14.43	N/A

▼ Formula Finder Results

Name	Formula	Score	m/z (Da)	Error (ppm)	Error MSMS (ppn

▼ Library Search Results

Name	CAS#	Formula	MM (Da)	Fit	Rev. Fit	Purity	CE (eV

Maesil_x100 - Citric acid (Unknown) 193..._pos_50-1250.wiff2), (sample Index: 2)
Area: 8.780e5, Height: 1.926e5, RT: 14.41 min

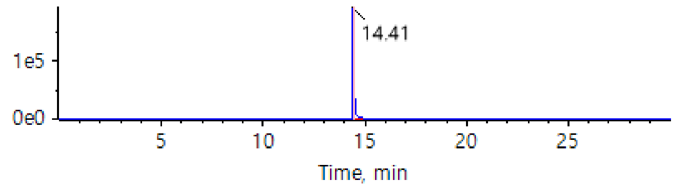

Spectrum from IHnE_IDA screening_... 1250) from 14.381 to 14.433 min
[C6H8O7+H]+

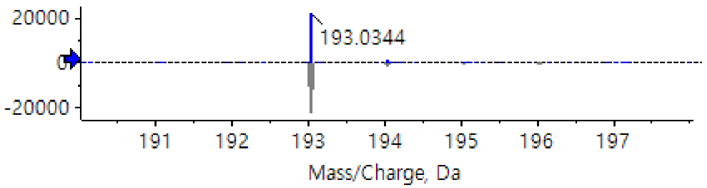

Spectrum from IHnE_IDA screening_p...193.0 Da, +1, CE: 35.0, CES: 15.0
Library Spectrum: Citric acid (HR NIST) (77929) , CE=20±0

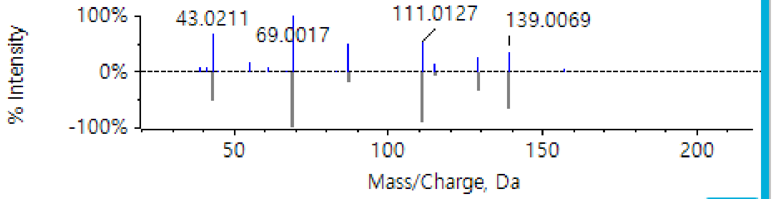

▼ Peak Details

Precursor m/z	Mass Error (ppm)	Retention Time (min)	Ion Ratio
193.0343	0.9	14.41	N/A

▼ Formula Finder Results

Name	Formula	Score	m/z (Da)	Error (ppm)	Error MSMS (ppn

▼ Library Search Results

Name	CAS#	Formula	MM (Da)	Fit	Rev. Fit	Pur
Citric acid (HR NIST)	77929	C6H8O7	192.027	100.0	99.7	99.7

Citric acid and its Derivatives, Metabolites

DL-Isocitric acid and lactone ($M+H_2O$)

Index	Sample Name	Component Name	Expected RT	Area	Adduct / Charge	Formula	Precursor Mass	Found At Mass	Mass Error (ppm)	Mass Error...	Isoto... Confi...	Library Confi...	Library Hit	Library Score	Isotope Ratio...	U...
2	BK_DW_1uL	DL-Isocitric acid...	14.43	3.448e3	[M-H]-	C6H6O6H...	191.0197	191.0186	-5.8	▲	▲	●	(2Z)-2-Phenyl-2-butenedioic acid (HR NIST) [Smart C...	29.9	9.9	☑
4	Maesil_x100	DL-Isocitric acid...	10.34	1.254e5	[M-H]-	C6H6O6H...	191.0197	191.0191	-3.5	✔	✔	✔	DL-Isocitric acid lactone (HR NIST)	91.4	0.9	☑

BK_DW_1uL - DL-Isocitric acid lactone (...g_neg_50-1250.wiff2), (sample Index: 2)
Area: 3.448e3, Height: 7.003e2, RT: 14.43 min

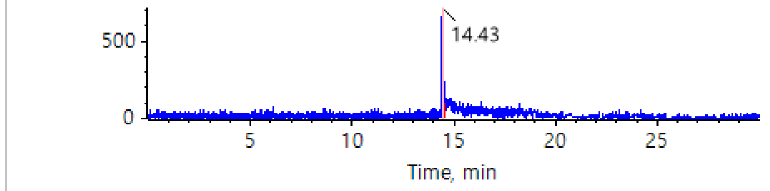

Spectrum from IHnE_IDA screening_... 1250) from 14.411 to 14.462 min
[C6H6O6H2O-H]-

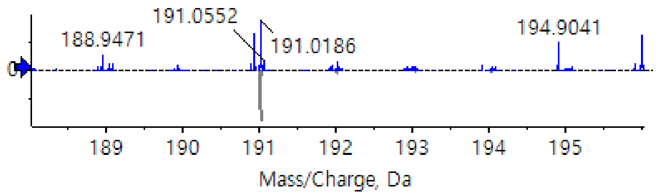

Spectrum from IHnE_IDA screening_n...191.0 Da, -1, CE: -35.0, CES: 15.0
Library Spectrum: (2Z)-2-Phenyl-2-b...acid (HR NIST) (16110988) , CE=24±0

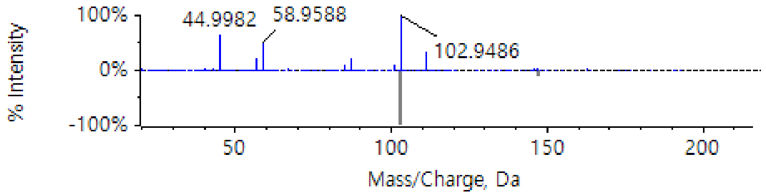

Peak Details

Precursor m/z	Mass Error (ppm)	Retention Time (min)	Ion Ratio
191.0197	-5.8	14.43	N/A

Formula Finder Results

Name	Formula	Score	m/z (Da)	Error (ppm)	Error MSMS (ppn

Library Search Results

Name	CAS#
(2Z)-2-Phenyl-2-butenedioic acid (HR NIST) [Smart Confirmation]	16110988

Maesil_x100 - DL-Isocitric acid lactone ...g_neg_50-1250.wiff2), (sample Index: 3)
Area: 1.254e5, Height: 6.812e3, RT: 10.34 min

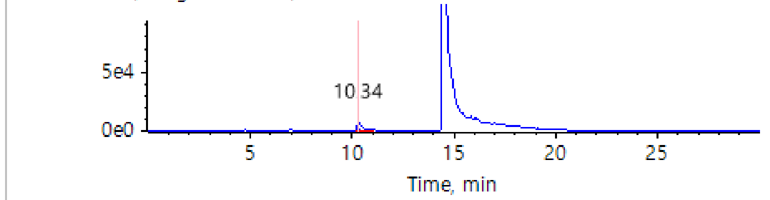

Spectrum from IHnE_IDA screening_... 1250) from 10.324 to 10.375 min
[C6H6O6H2O-H]-

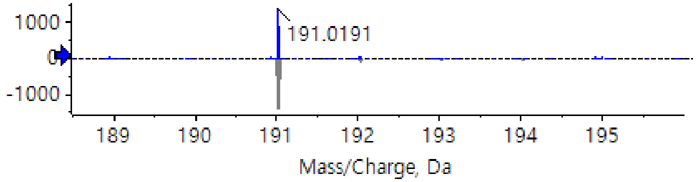

Spectrum from IHnE_IDA screening_n...191.0 Da, -1, CE: -35.0, CES: 15.0
Library Spectrum: DL-Isocitric acid lactone (HR NIST) (4702323) , CE=21±0

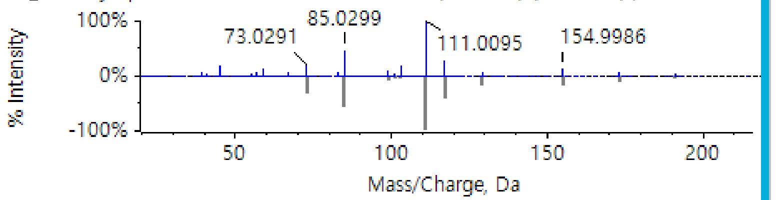

Peak Details

Precursor m/z	Mass Error (ppm)	Retention Time (min)	Ion Ratio
191.0197	-3.5	10.34	N/A

Formula Finder Results

Name	Formula	Score	m/z (Da)	Error (ppm)	Error MSMS (ppn

Library Search Results

Name	CAS#	Formula	MM (Da)	Fit
DL-Isocitric acid lactone (HR NIST)	4702323	C6H6O6	174.01644	98.2

Additional components

3-O-Coumaroylquinic acid

Index	Sample Name	Component Name	Expected RT	Area	Adduct / Charge	Formula	Precursor Mass	Found At Mass	Mass Error (ppm)	Mass Error...	Isoto... Confi...	Library Confi...	Library Hit	Library Score	Isotope Ratio...	U...
3	BK_DW_1uL	3-O-Coumaroyl...	16.87	N/A	[M-H]-	C16H18O8	337.0929	N/A	N/A	■	■	■		N/A	N/A	☑
6	Maesil_x100	3-O-Coumaroyl...	16.87	1.635e5	[M-H]-	C16H18O8	337.0929	337.0915	-4.3	✓	✓	✓	3-O-Coumaroylquinic acid (HR NIST)	100.0	3.5	☑

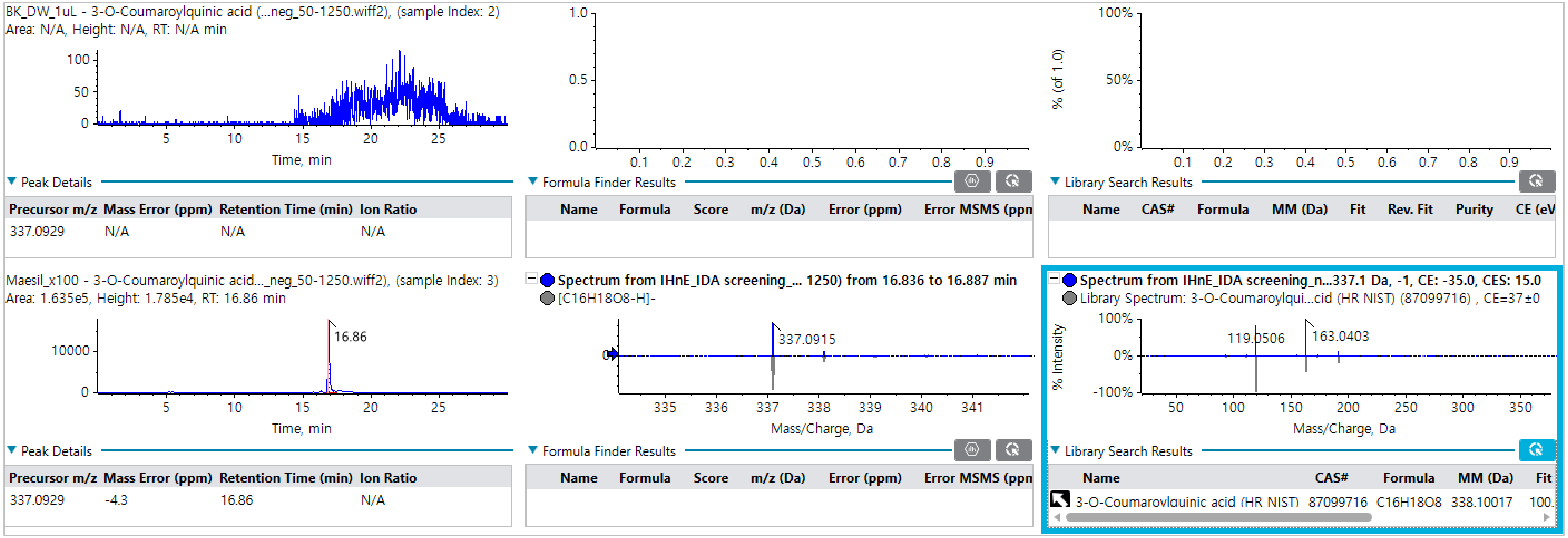

Additional components

Eugenol gentiobioside

Index	Sample Name	Component Name	Expected RT	Area	Adduct / Charge	Formula	Precursor Mass	Found At Mass	Mass Error (ppm)	Mass Error...	Isoto... Confi...	Library Confi...	Library Hit	Library Score	Isotope Ratio...	U...
5	BK_DW_1uL	Eugenol gentio...	17.45	N/A	[M+NH4]+	C22H32O12	506.2232	N/A	N/A					N/A	N/A	☑
10	Maesil_x100	Eugenol gentio...	17.45	1.580e4	[M+NH4]+	C22H32O12	506.2232	506.2236	0.8	✓	✓	✓	Eugenol gentiobioside (HR NIST)	98.0	1.4	☑

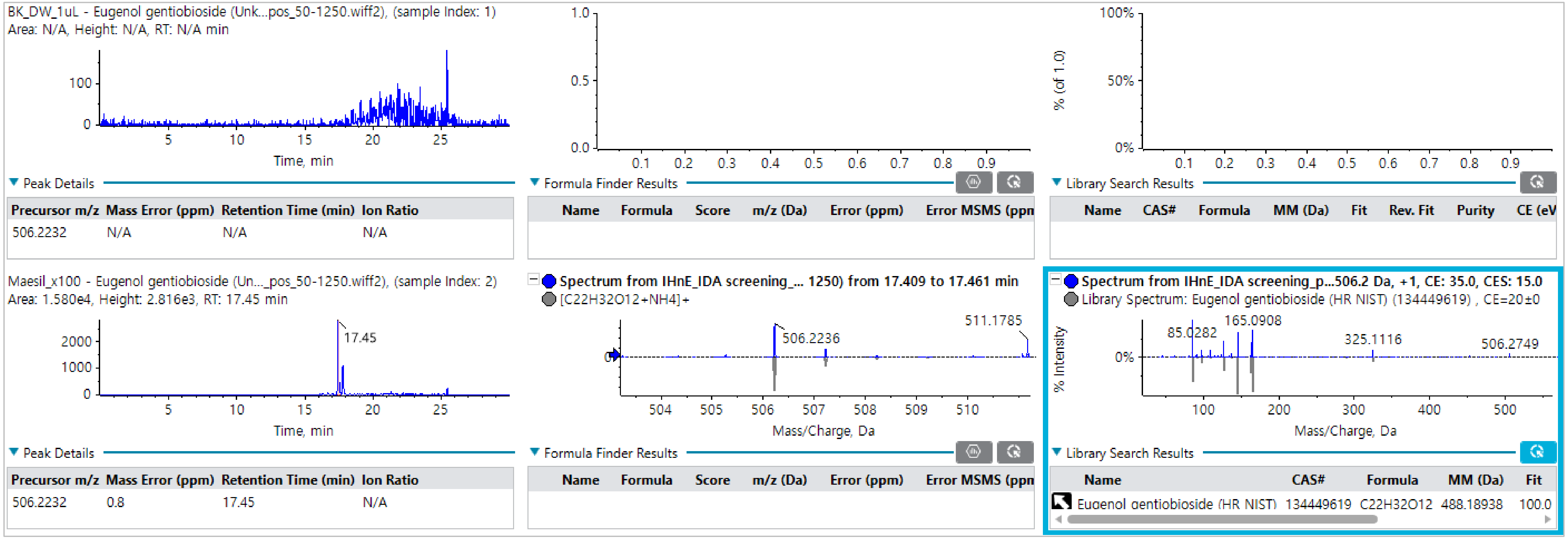

Additional components

Flavovilloside

Index	Sample Name	Component Name	Expected RT	Area	Adduct / Charge	Formula	Precursor Mass	Found At Mass	Mass Error (ppm)	Mass Error...	Isoto... Confi...	Library Confi...	Library Hit	Library Score	Isotope Ratio...	U...
6	BK_DW_1uL	Flavovilloside	16.49	N/A	[M+H]+	C33H40O20	757.2186	N/A	N/A	■	■	■		N/A	N/A	☑
12	Maesil_x100	Flavovilloside	16.49	1.359e5	[M+H]+	C33H40O20	757.2186	757.2192	0.8	✓	✓	✓	Flavovilloside (HR NIST)	96.7	4.4	☑

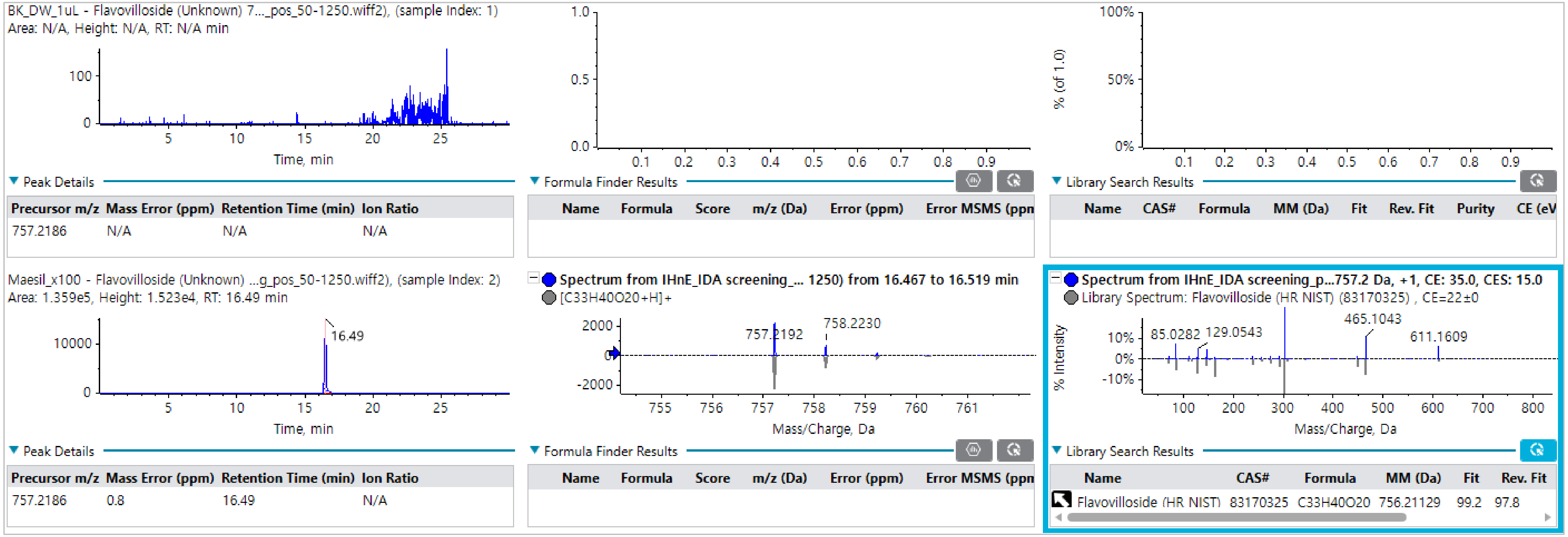

Additional components

Abscisic acid

Index	Sample Name	Component Name	Expected RT	Area	Adduct / Charge	Formula	Precursor Mass	Found At Mass	Mass Error (ppm)	Mass Error...	Isoto... Confi...	Library Confi...	Library Hit	Library Score	Isotope Ratio...	U...
7	BK_DW_1uL	Abscisic acid	19.54	1.844e3	[M+H]+	C15H18O3	247.1329	247.1337	3.3	✓	●	■	No Acquired MSMS	N/A	99.7	☑
14	Maesil_x100	Abscisic acid	19.54	3.644e4	[M+H]+	C15H18O3	247.1329	247.1327	-0.8	✓	✓	✓	(+)-Abscisic acid (HR NIST) [Smart Confirmation]	81.5	1.8	☑

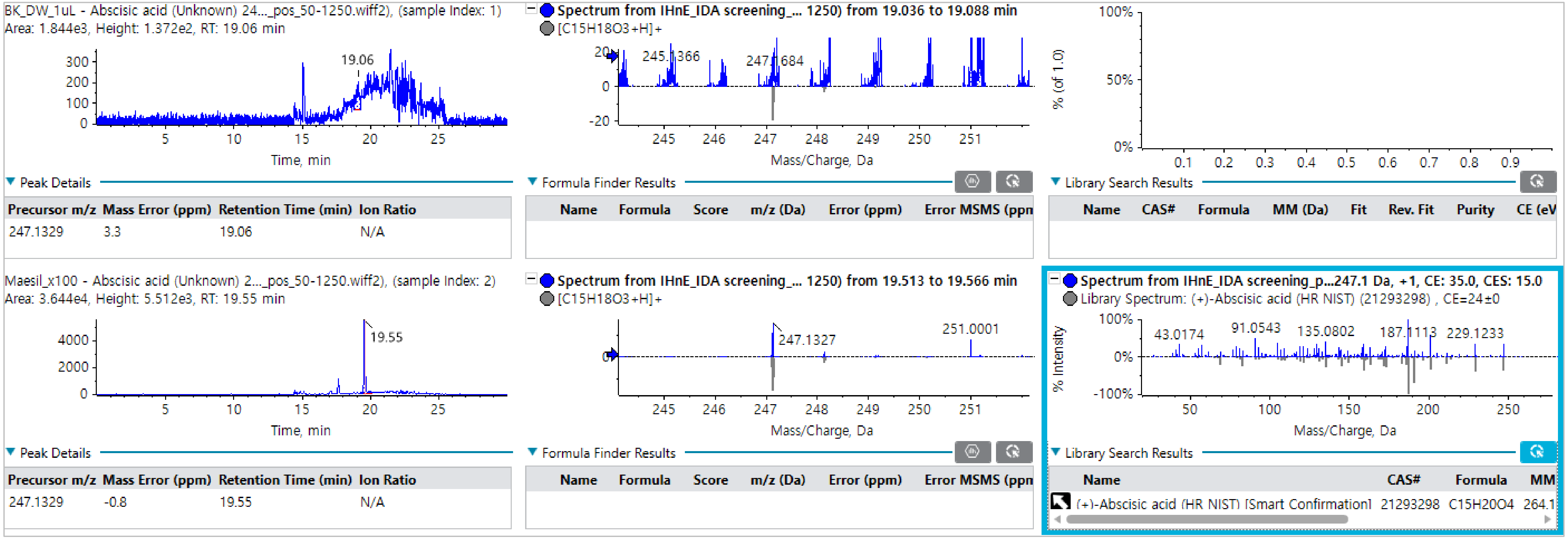

Supplement: Supplementary file 1 [file molecules-31-03786-s001.zip › Supplementary spectra.pdf]
